# Mechanochemical transformation of planar polyarenes to curved fused-ring systems

**DOI:** 10.1038/s41467-021-25495-6

**Published:** 2021-08-31

**Authors:** Teoh Yong, Gábor Báti, Felipe García, Mihaiela C. Stuparu

**Affiliations:** Division of Chemistry and Biological Chemistry, School of Physical and Mathematical Sciences, Nanyang Technological University, Singapore, Singapore

**Keywords:** Solid-phase synthesis, Synthetic chemistry methodology, Organic molecules in materials science

## Abstract

The transformation of planar aromatic molecules into π-extended non-planar structures is a challenging task and has not been realized by mechanochemistry before. Here we report that mechanochemical forces can successfully transform a planar polyarene into a curved geometry by creating new C-C bonds along the rim of the molecular structure. In doing so, mechanochemistry does not require inert conditions or organic solvents and provide better yields within shorter reaction times. This is illustrated in a 20-minute synthesis of corannulene, a fragment of fullerene C_60_, in 66% yield through ball milling of planar tetrabromomethylfluoranthene precursor under ambient conditions. Traditional solution and gas-phase synthetic pathways do not compete with the practicality and efficiency offered by the mechanochemical synthesis, which now opens up a new reaction space for inducing curvature at a molecular level.

## Abstract (web summary)

The transformation of planar aromatic molecules into non-planar structures is a challenging task and has not been realized by mechanochemistry before. Here, the authors demonstrate that mechanochemical forces can transform a planar polyarene into a curved geometry by creating new C-C bonds along the rim of the molecular structure.

## Introduction

The synthesis of strained aromatic molecules from strain-free precursors is a challenging task. This requires the natural trigonal planar geometry of the *sp*^*2*^-hybridised carbon atoms to become non-planar. The angle strain associated with this pyramidalization needs to be overcome in any viable synthesis. This necessitates application of either high-energy reaction conditions or high-energy precursors. For instance, flash vacuum pyrolysis (FVP), in which molecules are subjected to high temperatures (500–1100 °C) in the gas-phase, is the most successful synthetic methodology to create non-planar molecules^1,2^. In a landmark publication in 1991, Scott established the utility of FVP as a synthetic method that could bend diethynylfluoranthene, a flat polyarene, into a bowl-shaped aromatic structure, corannulene^3^. Scott reasoned that the high-energy conditions temporarily populate bent molecular geometries that would be inaccessible under normal circumstances. Only under such folded conformation, the two-carbon side chains could reach across the fluoranthene bay region to intramolecularly generate two new 6-membered rings. Ever since, a large family of molecular bowls have been produced through this fascinating method^1^. A decade later the technique reached a new high in the chemical synthesis of fullerene C_60_^4,5^. This feat of organic synthesis serves as an inspiration to explore new methods that can twist aromatic molecules and create new covalent bonds to lock the curved molecular geometry.

**Fig. 1:**
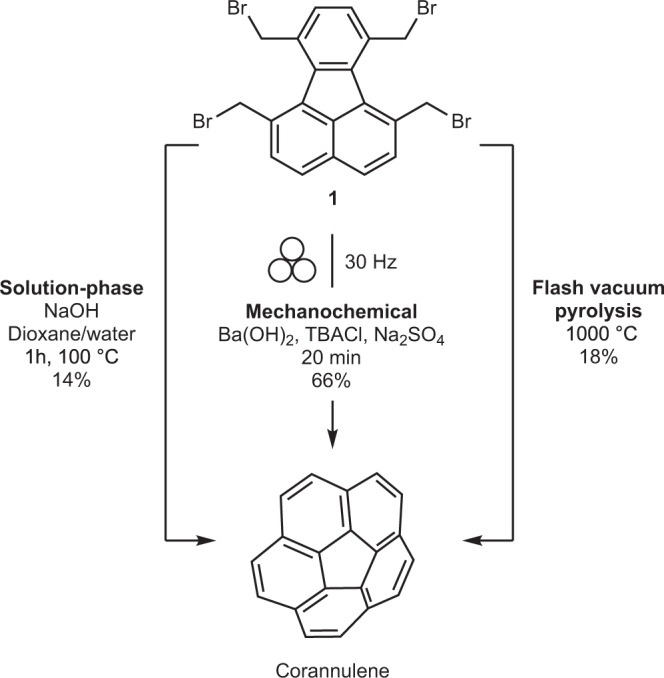
Comparison of different synthetic pathways to corannulene. A comparison of the gas-phase, solid-phase and liquid-phase synthesis of corannulene.

Mechanochemistry, the use of mechanical force to drive chemical reactions, promises sustainable, faster, scalable and efficient processes^6–21^. In this regard, in the past few years, various synthetic techniques such as direct mechanocatalysis^21–25^ and liquid assisted grinding^26–31^ are developed. New possibilities for heating^32–34^, cooling^35,36^ and conducting gaseous^37,38^ mechanochemical reactions are established. Furthermore, analytical techniques for in situ monitoring of the reactions are described^39–45^. Along with these developments, the application of mechanochemistry for the synthesis of various polyarenes has also been increasing^46–51^. So far, however, mechanochemistry is not known to transform planar aromatic molecules into non-planar structures. We reasoned that mechanochemistry, similar to FVP, creates extraordinary reaction conditions but through impact and shear forces. Such conditions maybe able to bend the molecules such as fluoranthene and allow them to react at the bay regions to form the curved fused-ring system. Unlike FVP, however, mechanochemistry offers no restrictions on the nature of precursors (flash vacuum pyrolysis requires the precursor to be able to sublime and withstand the sublimation conditions), a simpler experimental set-up, possible scalability, better yields, shorter times and operation under ambient conditions. Therefore, it might be a valuable method to access curved structures. To explore this hypothesis, we focused our efforts on tetrabromofluoranthene (**1**), as a precursor to corannulene synthesis (Fig. 1)^52^. This allows for a direct comparison to be made not only with the gas-phase FVP^53^ but also the conventional solution-phase synthesis^54^.

In this work, we show that mechanochemistry is indeed capable of inducing curvature at a molecular level. It is also a practically simple, mild, fast, high-yielding and a sustainable synthetic approach.

## Results and discussion

### Synthesis of planar polyarene precursor

Initially, we focused on the synthesis of precursor **1**. The goal was to explore an environmentally friendly approach to access **1**. For this, our investigations began with a key compound, 3,8-dimethylacenaphthenequinone **2**, that can be procured from commercial sources. An aldol condensation of **2** leads to **3**, followed by a Diels-Alder reaction to yield 1,6,7,10-tetramethylfluoranthene **4**. Tetramethylfluoranthene **4** then undergoes benzylic bromination to form precursor **1**.

**Fig. 2:**
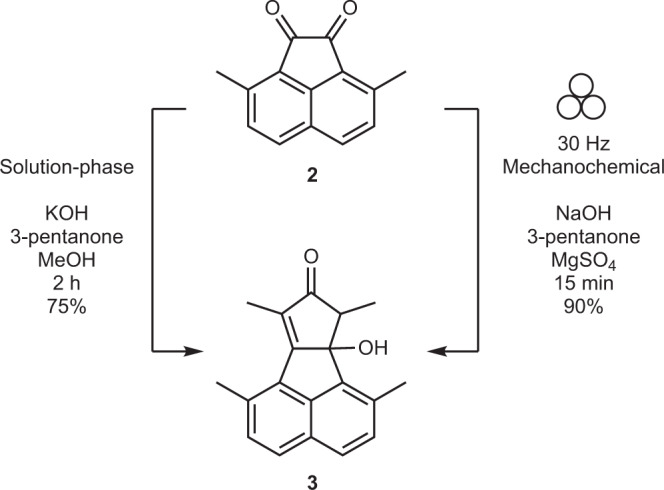
Synthesis of 3. The solution-phase reaction was reproduced using the procedure provided in Butterfield et al.^55^.

The aldol condensation of 3,8-dimethylacenaphthenequinone **2** with 3-pentanone was carried out under basic conditions (Fig. 2). Solution-based method requires 22 equiv of KOH dissolved in methanol to attain a sufficiently high pH for the reaction^55^. The excess of base must then be tediously neutralised using HCl to precipitate the product. Excessive acidification causes product **2** to irreversibly dimerise to **3a** (Supplementary Fig. 1).

Mechanochemistry circumvents these issues as the reaction is complete within 15 min of ball milling using just 2.5 equiv of NaOH as the base, and MgSO_4_ as the grinding auxiliary. The crude product requires no chromatographic purification. The reaction is easily scalable to 1 g with no notable difference in isolated yields. Investigations into the reaction conditions identify MgSO_4_ as an essential grinding auxiliary, likely serving as both a dehydrating agent and a Lewis acid for the aldol condensation (Supplementary Table 1). NaOH can be replaced with a milder base such as K_2_CO_3_. The reaction progresses in this case too, albeit at a slower rate and requires two millings of 15 min each for completion (Supplementary Table 1). As a control, an un-milled reaction mixture with NaOH was stirred with a stir bar with no visible product formation observed after 30 min. This persists despite heating the reaction mixture to 50 °C. This contrast in reactivity suggests that ball milling is responsible for the mechanochemical activation of the reaction. Overall, ball milling makes the reaction greener and simplifies the workup by removing the laborious neutralisation step of excess NaOH, greatly reducing reaction time and generating cleaner products in higher yields.

**Fig. 3:**
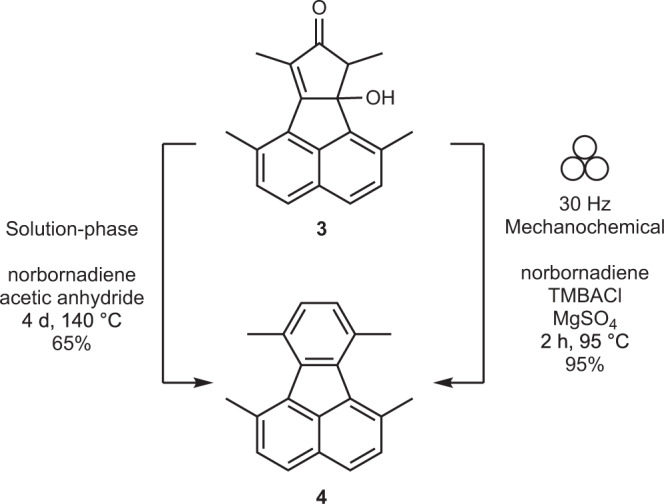
Synthesis of 4. The solution-phase reaction was reproduced using the procedure provided in Butterfield et al.^55^.

In the next step, compound **3** undergoes an inverse electron demand Diels-Alder cheletropic elimination followed by a retro Diels-Alder reaction cascade to generate tetramethylfluoranthene **4** (Fig. 3). Initial milling of compound **3** with MgSO_4_ and norbornadiene at room temperature generates the dimer **3b** only (Supplementary Fig. 1). This is likely due to the high activation energy required by the Diels-Alder reaction between norbornadiene and **3**. This is verified by the successful Diels-Alder reaction of **3** with a more reactive dienophile, dimethylacetylene dicarboxylate (Supplementary Fig. 1 and Supplementary Table 2). Therefore, jars were heated to 95 °C^51^ during the milling process to attain the required activation energy^56^. This change in milling conditions drives the reaction to completion within 2 h. In comparison, solution-phase synthesis requires stirring for 4 days at 140 °C to produce 65% of **4**. It is important to note that snap-fit jars were used which allowed the norbornadiene to escape. If a screw-tight jar is used, the yield is halved due to high pressure inhibiting the chelotropic elimination of CO gas, which is part of the reaction cascade.

**Fig. 4:**
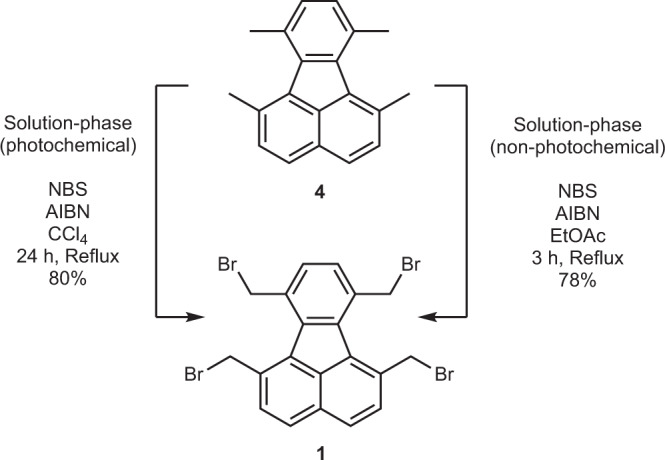
Synthesis of precursor 1. The reaction conditions for the solution-phase photochemical synthesis is adopted from Borchardt et al.^53^.

Mechanochemical benzylic bromination of **4** proved to be the most demanding step in this synthesis as ball milling strongly favours aromatic bromination over benzylic bromination, with no literature reported on mechanochemical Wohl–Ziegler bromination. Therefore, other advances in green chemistry were considered in favour of mechanochemistry for the synthesis of **1** (Fig. 4).

A user and environmentally friendly alternative is using ethyl acetate as the solvent^57^. Although the mechanism of the reaction is still unknown, ethyl acetate has been proven to be effective for benzylic bromination even in the absence of light^57^. Although initial reflux in ethyl acetate only generates a complex mixture of mono and dibrominated products, the addition of azobisisobutyronitrile (AIBN) as a free-radical source resolves this, generating tetrabromomethylfluoranthene within 3 h (Supplementary Table 3). The crude product can be subjected to the next reaction. However, to achieve better results, a pure product can be isolated at 78% yield by recrystallisation with ethyl acetate. In comparison, photochemical synthesis requires a light source, an excess of NBS, and provides 80% yield in 24 h of reaction time in toxic CCl_4_^53^.

### Inducing molecular curvature

Having efficient access to precursor **1**, we began to investigate the possibility of intramolecular formation of aromatic rings at the bay region of the fluoranthene nucleus. For this, we chose to employ basic conditions which is known to produce corannulene from precursor **1** in solution albeit with low yields^54^. In solid-phase too, only low yields of corannulene (2–5%) could be obtained upon milling of **1** with NaOH or NaO*t*-Bu (Supplementary Table 4). We attributed these poor results to essentially a bi-phasic system in which the inorganic base and organic reactant did not interact with each other. To improve contact between reactants, we looked towards traditional phase-transfer agents such as tetrabutylammonium chloride (TBACl). The hygroscopic nature of TBACl was observed to turn the crude reaction mixture into a gooey solid. We observed that 5 min of pre-milling of the precursor with TBACl followed by a 15 min of milling with barium hydroxide produced the best results and provided corannulene in 66% isolated yield. Without pre-milling step, the milling times of 10 and 20 min provided comparatively lower yields of 64 and 60%, respectively. In terms of total reaction time, the reaction with 5 min of pre-mill and 15 min of milling (yield = 66%) provides a comparison with the 20 min reaction (yield = 60%). It is reasonable to assume that a longer exposure of the reactants and the product to the strong basic conditions favour formation of side products and lowers corannulene yield. Finally, we observed that the reaction failed to produce any corannulene in the absence of a base (Supplementary Table 4).

**Fig. 5:**
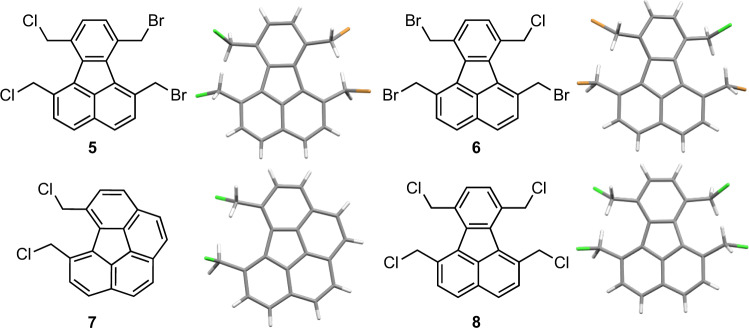
Chemical and X-ray crystal structures of 5, 6, 7 and 8. The structures for **5** and **6** are found to be disordered at the halogen atoms due to cocrystallization of compounds carrying bromide (shown with a green colour) and chloride (shown with a brown colour) substituents.

To examine whether TBACl could be replaced with similar other salts, tetrabutylammonium bromide (TBAB) and tetrabutylammonium fluoride (TBAF) were used. However, this led to a drastic reduction in yield (5%) or a complete failure of reaction (Supplementary Table 4). This suggested that simply a better mixing of solids was not the sole reason behind better yields in the presence of TBACl. To investigate whether TBACl participated in the reaction, pre-mill stage solid was analysed (before addition of a base) and found to contain a fluoranthene derivative **5** in which two bromine atoms were replaced by two chlorine atoms (Fig. 5). This result meant that TBACl was involved in the reaction. Intrigued by this, we began to examine whether byproducts could be isolated from corannulene synthesis. Fortunately, we isolated two trace compounds which were confirmed to be compounds **6** and **7** through X-ray crystallography (Fig. 5). Overall, therefore, it appears that during the reaction, the nucleophilic chloride anions displace the bromine atoms from precursor **1**. A base is necessary for the reaction, and the more robust and more electronegative chlorine atoms would stabilise the benzylic cabanions more efficiently. Therefore, it is reasonable to assume that besides improving mixing of solids, TBACl acts as a chlorinating agent and the reaction mechanism involves anionic intermediates.

**Fig. 6:**
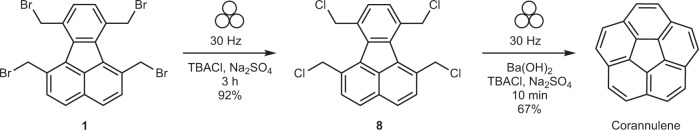
A new synthetic route to corannulene. Synthesis of corannulene from tetrachloromethylfluoranthene precursor **8**.

The finding that chlorinated compounds were involved in the reaction arose an interesting possibility: can tetrachloromethylfluoranthene (**8**) serve as a precursor for corannulene synthesis? To answer this, we employed the knowledge gained thus far to prepare **8** through subjecting tetrabromomethylfluoranthene to a mechanochemical halide-exchange reaction with the help of TBACl (Fig. 6). This reaction produced **8** in an isolated yield of 92%. Precursor **8** was then subjected to the ball milling reaction under identical conditions as in the case of **1** and produced the best yield of 67% of corannulene. TBACl was, however, still required for a successful reaction indicating that the mixing of solids was also critical to the success of the reaction (Supplementary Table 4). Furthermore, since chloride precursor **8** adds an additional step to the synthesis, the bromide precursor **1** still represents an optimum route to corannulene synthesis. In comparison to the solid-phase synthesis, FVP at 1000 °C provides 18%^53^ while Sygula’s solution-phase synthesis gives an isolated yield of 14% of corannulene from precursor **1**^54^.

### Mechanistic aspects

**Fig. 7:**
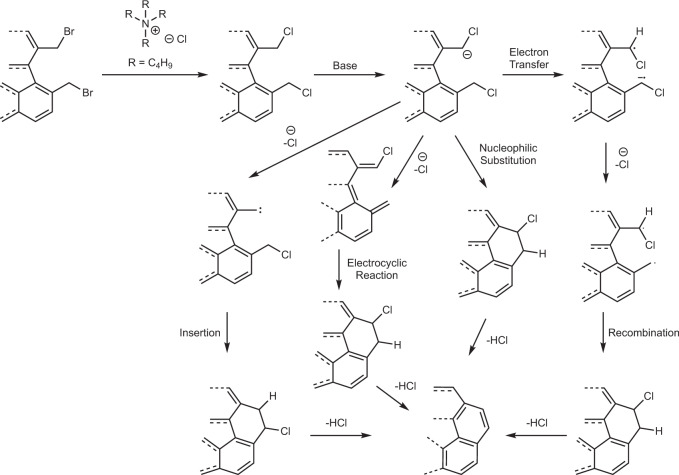
Mechanistic pathways to corannulene. Plausible mechanistic pathways in the transformation of **1** to corannulene. The positions of Cl/H are tentative. Only a partial structure is shown for simplicity reasons.

In terms of reaction mechanism, based on the formation of compounds **5**–**7** during synthesis, we assume that bromine atoms are first replaced with chlorine atoms. Subsequently, benzylic protons are deprotonated since the reaction does not work in the absence of a base. The support for this notion comes from the work of Kharasch who established coupling of benzyl halides in the presence of a strong base^58^. Later work in the area by Dubios and Gingras showed that intramolecular ring formation through carbenoid coupling could yield phenanthrene and [5]-helicene structures beginning with benzyl halide precursors^59^. While the exact reaction mechanism for such processes still remains unknown, the proposed pathways include nucleophilic substitution, electrocyclic reactions, carbene formation by α-elimination of HCl and radical-mediation (Fig. 7). It is difficult to differentiate between these pathways as they lead to the same product.

### Absorption characteristics

**Fig. 8:**
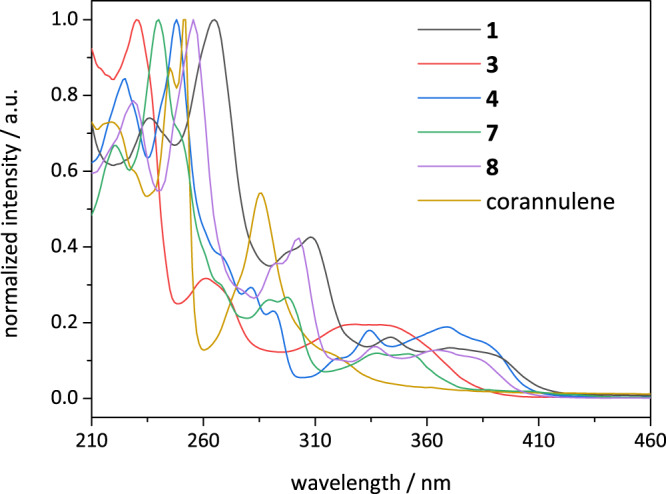
Absorption characteristics of the synthesised compounds. UV-Vis spectra of compounds **1** (black line), **3** (red line), **4** (blue line), **7** (green line), **8** (violet line) and corannulene (orange line) in acetonitrile at room temperature.

In UV-Vis spectroscopy (Fig. 8), compound **3** displays three broad absorption bands at 230, 261 and 334 nm. Upon Diels-Alder reaction, which forms the fluoranthene nucleus (**4**), a bathochromic shift of 34 nm is observed due to extension in π-conjugation. Fluoranthene absorption is known to be highly complex with a number of independent electronic transitions^60^. This can be observed in the case of halide derivatives **1** and **8** with absorption extending into the blue region of the electromagnetic spectrum. The halide substitution pattern does not alter the absorption characteristics due to a methylene spacer, which interrupts the electronic conjugation between the halides and the aromatic nucleus. Formation of the first aromatic ring in the bay region of fluoranthene, however, results in loss of molecular planarity and a blue shift of ~25 nm is observed for compound **7**. Formation of a strained bowl upon second ring-closure results in a further loss in planarity and a further blue shift of 57 nm. The absorption spectrum of corannulene matches well with the previous literature reports^61,62^.

### Comparison with solution-phase synthesis

**Fig. 9:**
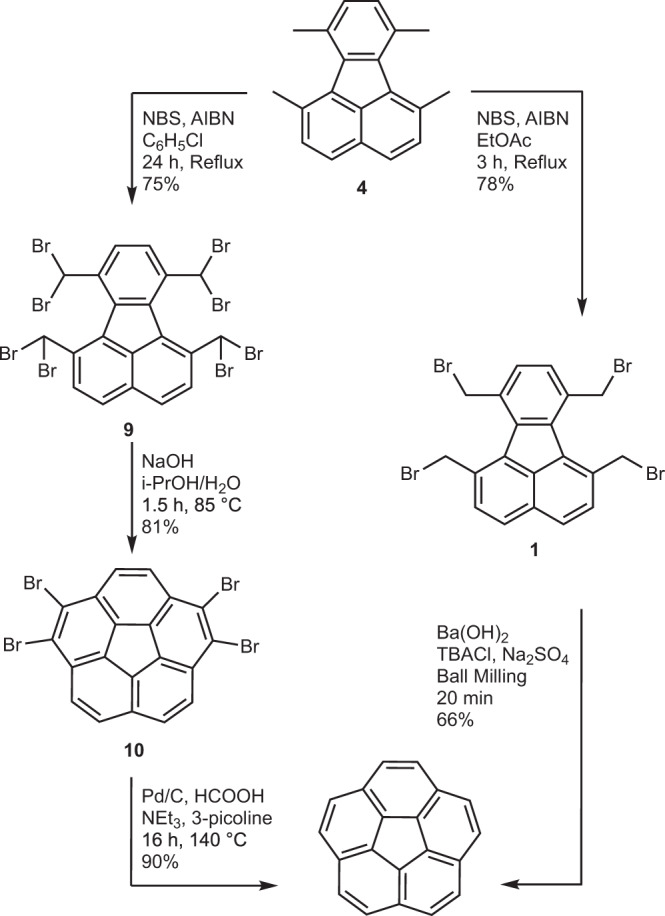
Comparison of solution and mechanochemical synthetic pathways to corannulene. A comparison of the present synthesis (right) with the optimized kg scale solution-phase synthesis (left) of corannulene reproduced using the procedures provided in Butterfield et al.^55^.

Finally, a comparison can be made with an optimised solution-phase synthesis of corannulene^55^ which employs the high-energy octabrominated precursor **9** (Fig. 9 and Supplementary Fig. 2)^63^. The excessive bromination in **10** means that an additional debromination step is required to obtain corannulene. Compared to this optimized synthesis of corannulene, the present synthesis, beginning with dimethylacenaphthenequinone, improves overall yield by 17%, reduces overall reaction time from a few days to a few hours, and reduces the amounts of the reagents required (Supplementary Fig. 2). Finally, it eliminates the need for the environmentally harmful solvents such as chlorobenzene from corannulene synthesis.

In summary, mechanochemistry offers a promising alternative to conventional gas-phase and solution-based synthetic methods in inducing molecular curvature. With the help of corannulene, it can be demonstrated that the solid-phase synthesis is practically simple. It can be carried out under ambient conditions and requires a shorter reaction time while producing a higher yield. Unlike FVP, it does not require a volatile precursor or high-energy conditions. Unlike solution-phase synthesis, it does not require a high-energy precursor or solvents. Overall, therefore, given the potential of scalability and the challenges in developing high-yielding sustainable synthesis of curved fused-ring systems, mechanochemistry appears to be a worthy alternative to the traditional solution and gas-phase chemistries.

## Methods

### Synthesis of 3

1.0 g 3,8-Dimethylacenaphthenequinone (4.76 mmol), 476 mg NaOH (11.9 mmol), 3-pentanone (9.52 mmol, 0.8 g), MgSO_4_ (19.04 mmol, 2.6 g) and a 15 mm ∅ stainless steel ball (13.55 g) were added to a 30 mL stainless steel jar. The jar was tightly sealed using parafilm and milled at 30 Hz for 15 min. The resulting powder was scraped from the jar, dispersed in 10 mL of acetone and filtered. The filtered cake was washed twice with 10 mL of acetone and the filtrate is evaporated to yield **3** (1.2 g, 90% yield). ^1^H NMR (400 MHz, CDCl_3_): δ 7.76 (d, *J* = 8.3 Hz, 1H), 7.67 (d, *J* = 8.3 Hz, 1H), 7.36 (dd, *J* = 14.4, 8.3 Hz, 2H), 2.90 – 2.75 (m, 4H), 2.61 (s, 3H), 2.15 (s, 3H), 1.68 – 1.61 (m, 3H). ^13^C NMR (100 MHz, CDCl_3_): δ 210.97, 170.65, 139.26, 139.12, 133.36, 131.89, 131.00, 130.92, 130.87, 130.79, 130.78, 130.73, 128.34, 128.06, 125.82, 86.19, 48.82, 22.23, 19.87, 11.21, 10.34. HRMS (EI) calcd for C_19_H_18_O_2_ (M+) 278.1307, found 278.1311.

### Synthesis of 4

100 mg of **3** (0.36 mmol), 294 µL norbornadiene (2.88 mmol), 131 mg trimethylbenzylammonium chloride (0.72 mmol), 68 µL acetic anhydride (0.72 mmol) and 600 mg of MgSO_4_ were loaded into a 15 mL stainless steel jar (10 mm Teflon ring) with a 10 mm ∅ stainless steel ball (4.02 g). The jar is heated to 95 °C and milled at 30 Hz for 2 h, with 20 min break in between. The crude mixture is scrapped from the jar and purified using a short silica plug (eluent: 100% hexane) to obtain **4** as a yellow solid (88 mg, 95% yield). ^1^H NMR (400 MHz, CDCl_3_): δ 7.82 (d, *J* = 8.2 Hz, 2H), 7.50 (d, *J* = 8.2 Hz, 2H), 7.26 (s, 2H), 2.99 (s, 6H), 2.90 (s, 6H). ^13^C NMR (100 MHz, CDCl_3_): δ 140.08, 135.05, 133.85, 132.15, 131.99, 130.83, 126.77, 126.31, 25.28, 24.46. HRMS (EI) calcd for C_20_H_18_ (M+) 258.1409, found 258.1406.

### Synthesis of 1

A 100 mL round bottom flask was charged with 1 g of **4** (3.88 mmol), 3.1 g (17.4 mmol) of *N*-bromo succinimide, 127 mg of AIBN (0.78 mmol) and 50 mL of ethyl acetate. A reflux condenser is attached and the mixture is heated to reflux for 3 h. The reaction mixture is cooled to room temperature and the solvent is removed under reduced pressure. The crude solid is then triturated in cold saturated aqueous NaHCO_3_ solution and filtered to remove succinimide. The residue is washed with saturated aqueous NaHCO_3_ solution followed by cooled methanol to obtain crude **1** as an orange solid. The crude sample can be used directly in the next step. However, to improve yield of corannulene synthesis, crude **1** is purified by recrystallisation from ethyl acetate to give a white powder (1.78 g, 78% yield). ^1^H NMR (400 MHz, CDCl_3_): δ 7.83 (d, *J* = 2.6 Hz, 2H), 7.72 (d, *J* = 8.5 Hz, 2H), 7.62 (d, *J* = 0.6 Hz, 2H), 5.00 (s, 3H), 4.96 (s, 3H). ^13^C NMR (100 MHz, CDCl_3_): δ 138.53, 133.66, 133.65, 133.28, 132.81, 132.68, 132.66, 129.41, 128.22, 34.38, 34.21. HRMS (EI) calcd for C_20_H_14_Br_4_ (M+) 573.7789, found 573.7766.

### Synthesis of corannulene from precursor 1 with pre-mill step

100 mg of **1** (0.17 mmol) is milled with 400 mg of tetrabutylammonium chloride (1.43 mmol) and 400 mg of sodium sulphate decahydrate in a 15 mL zirconia jar with a 10 mm ∅ zirconia ball (3.08 g) at 30 Hz for 5 min. 240 mg of barium hydroxide octahydrate (1.57 mmol) is added and the mixture is milled for 15 min at 30 Hz. The crude mixture was suspended in DCM and dry loaded onto silica gel. Column chromatography was performed (eluent: from 100% hexane to hexane:DCM 30:1) to obtain pure corannulene as a white powder (28 mg, 66% yield). ^1^H NMR (400 MHz, CDCl_3_): δ 7.80 (s, 10H). ^13^C NMR (100 MHz, CDCl_3_): δ135.90, 130.95, 127.15. HRMS (EI) calcd for C_20_H_10_ (M+) 250.0783, found 250.0782.

### Synthesis of corannulene from precursor 1 without pre-mill step

100 mg of **1** (0.17 mmol), 400 mg of tetrabutylammonium chloride (1.43 mmol), 400 mg of sodium sulphate decahydrate and 240 mg of barium hydroxide octahydrate (1.57 mmol) were loaded into a 15 mL zirconia jar with a 10 mm ∅ zirconia ball (3.08 g) and milled at 30 Hz for 10 min. The crude mixture was suspended in DCM and dry loaded onto silica gel. Column chromatography was performed (eluent: from 100% hexane to hexane:DCM 30:1) to obtain pure corannulene as a white powder (26 mg, 64% yield).

### Synthesis of 8

200 mg of **1** (0.34 mmol), 800 mg of tetrabutylammonium chloride (2.86 mmol) and 800 mg of sodium sulphate decahydrate were loaded into a two sets of 15 mL zirconia jars with a 10 mm ∅ zirconia ball (3.08 g) each and milled at 30 Hz for 3 h. The crude mixture was combined, suspended in water, filtered off and the solids were re-dissolved in DCM to dry load onto silica gel. Column chromatography was performed (eluent: from hexane:DCM 30:1 to hexane:DCM 20:1) to obtain pure tetrachloromethylfluoranthene **8** as an off-white solid (127 mg, 92% yield). ^1^H NMR (400 MHz, CDCl_3_): δ 7.86 (d, *J* = 8.5 Hz, 2H), 7.73 (d, *J* = 8.6 Hz, 2H), 7.62 (s, 2H), 5.06 (d, *J* = 10.4 Hz, 8H). ^13^C NMR (100 MHz, CDCl_3_): δ 138.73, 133.97, 133.66, 133.22, 132.45, 132.28, 132.17, 132.07, 129.47, 128.19, 45.55, 45.32. HRMS (EI) calcd for C_20_H_14_Cl_4_ (M+) 395.9822, found 395.9832.

### Synthesis of corannulene from precursor 8

43 mg of tetrachloromethylfluoranthene (0.11 mmol), 250 mg of tetrabutylammonium chloride (0.9 mmol), 250 mg of sodium sulphate decahydrate and 150 mg of barium hydroxide octahydrate (0.5 mmol) were loaded into a 15 mL zirconia jar with a 10 mm ∅ zirconia ball (3.08 g) and milled at 30 Hz for 10 min. The crude mixture was suspended in DCM and dry loaded onto silica gel. Column chromatography was performed (eluent: from 100% hexane to hexane:DCM 30:1) to obtain pure corannulene as a white powder (18 mg, 67% yield).

## Supplementary information

Supplementary Information

## Data Availability

All data supporting the findings of this study are available within the article and Supplementary information files, and also are available from the corresponding author upon reasonable request. The X-ray crystallographic coordinates for structures reported in this study have been deposited at the Cambridge Crystallographic Data Centre (CCDC), under deposition numbers CCDC 2085614 (corannulene), CCDC 2085628 (compound **5**), CCDC 2085612 (compound **6**), CCDC 2085613 (compound **7**) and CCDC 2085611 (compound **8**). These data can be obtained free of charge from The Cambridge Crystallographic Data Centre via www.ccdc.cam.ac.uk/data_request/cif.
