# Supplementary material for: Mechanochemical transformation of planar polyarenes to curved fused-ring systems
Source: Nat Commun. 2021 Aug 31;12:5187. doi: 10.1038/s41467-021-25495-6 (PMC8408202; doi:10.1038/s41467-021-25495-6)
Supplement: Supplementary file 1 — Supplementary Information [file 41467_2021_25495_MOESM1_ESM.pdf]

Mechanochemical transformation of planar polyarenes to curved fused-ring systems

Teoh Yong[1], Gábor Báti[1], Felipe García[1]*, and Mihaiela C. Stuparu[1]*

Division of Chemistry and Biological Chemistry, School of Physical and Mathematical Sciences, Nanyang Technological University, Singapore 21 Nanyang Link, 637371 Singapore.

*E-mail: fgarcia@ntu.edu.sg; mstuparu@ntu.edu.sg

Experimental Details

All reagents and solvents were obtained from commercial suppliers and used without further purification unless otherwise stated. 3,8-Dimethylacenaphthenequinone (1 g/10 $) was acquired from Hyderabad Laboratories (India). Mechanochemical reactions were carried out in stainless steel (30 mL and 15 mL, Form-Tech Scientific SmartSnap™), ZrO_2 (15 mL, Form-Tech Scientific SmartSnap™) or WC (15 mL Form-Tech Scientific SmartSnap™) grinder jars along with respective miller ball(s). The milling process was performed on Retsch Mixer Mill MM400 machine and subjected to 30 Hz milling for respective reaction time. TLC chromatograms (gel-coated aluminium plates, 60 F_{254}, Merck) were visualized with UV light (λ = 254 and 365 nm). Evaporation of organic solutions was achieved by rotary evaporation with a water bath temperature below 40 °C. Product purification by flash column chromatography was accomplished using Davisil® 40-63 micron silica gel. Technical grade solvents were used for chromatography and distilled prior to use. Compound **5** was obtained as a crude by suspending the pre-mill reaction mixture into water (to remove sodium sulphate and tetrabutylammonium chloride) and by washing the solid residue with dichloromethane to obtain the organic components. Compounds **6** and **7** were collected by column chromatography using a hexane to hexane:dichloromethane (20:1) gradient elution. All the crystals suitable for single crystal X-ray diffraction analysis were grown by slow evaporation from dichloromethane solutions at room temperature. The UV/Vis measurements were carried out on a Lambda 265 UV/Vis-spectrometer from Perkin-Elmer. HRMS was carried out on a JMS-700 from JEOL in EI mode. NMR spectra were recorded at room temperature on a 400 MHz and 500 MHz (and the corresponding frequencies for ^{13}C) Bruker and Jeol ECA NMR spectrometers. The residual solvent signals were taken as the reference (for $CHCl_3$ 7.26 ppm for ^1H NMR measurement and 77.23 ppm for ^{13}C NMR measurement). Chemical shifts are reported in delta (δ) units, parts per million (ppm) downfield from

trimethylsilane (TMS). Chemical shift (δ) is referred in terms of ppm, coupling constants (*J*) are given in Hz.

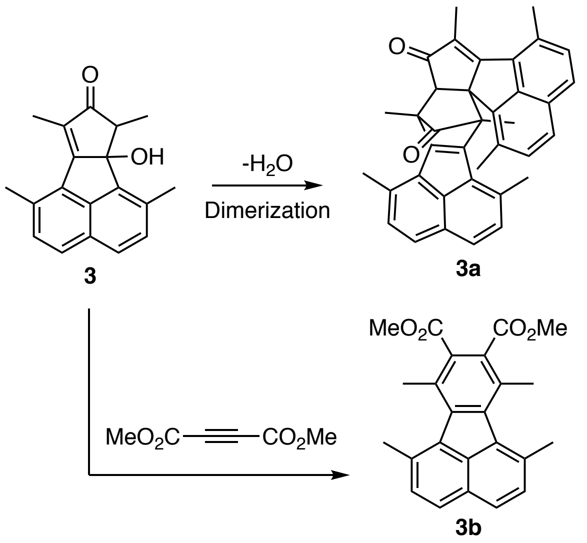

Supplementary Figure 1. Dimerization of **3** (top) and Diels-Alder reaction with dimethylacetylene dicarboxylate (bottom).

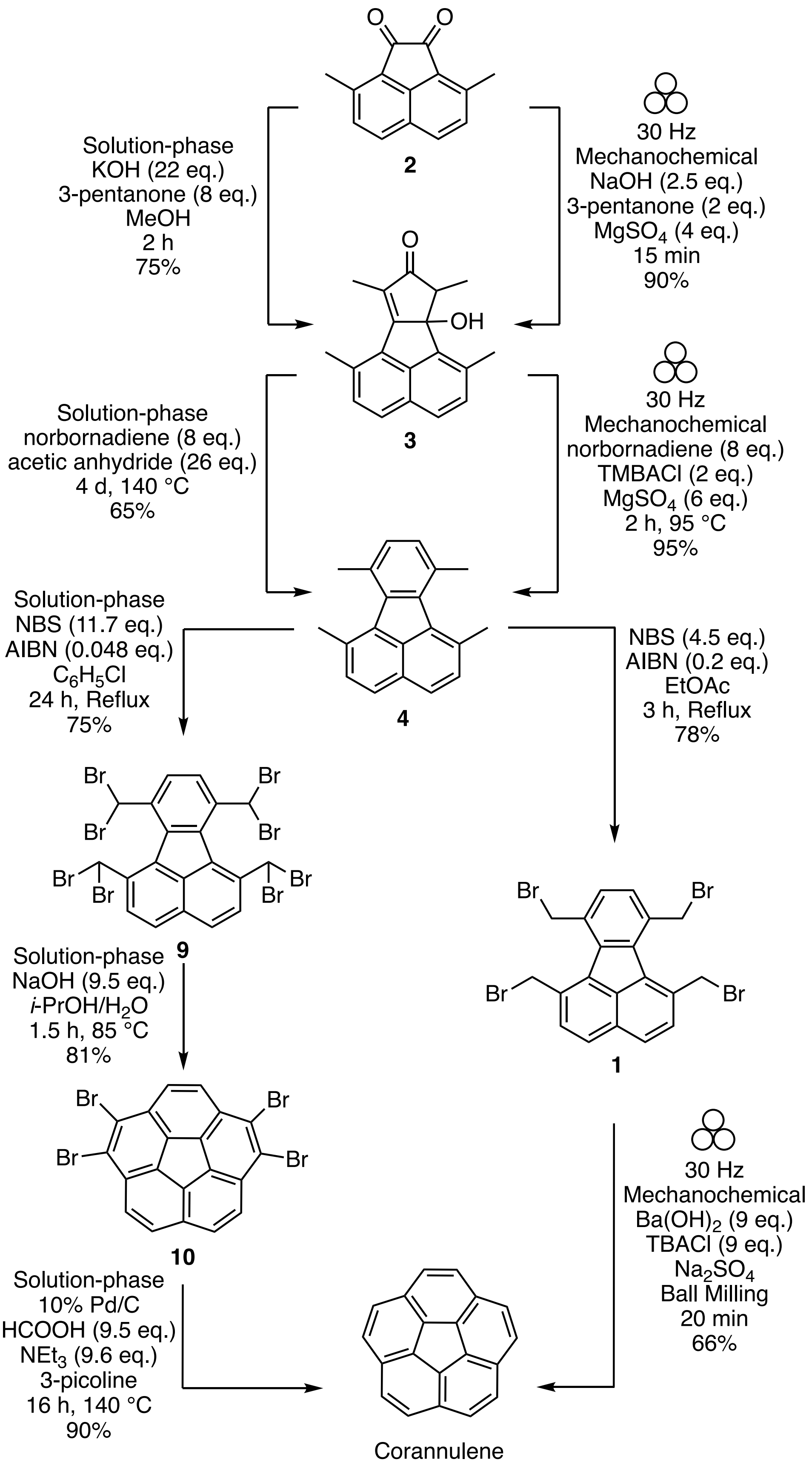

Supplementary Figure 2. A comparison of the optimized solution-phase (left) reproduced using procedures provided in reference 2 and currently developed (right) synthesis of corannulene.

Supplementary Table 1. Condition scans for the synthesis of 3.

Reaction conditions[a]				Results/Observation
**Milling time (Mins)**	**Base**	**Milling auxiliary**	**Resting time and temperature**	
30	2.5 eq. NaOH	Na_2SO_4	-	Trace product
30	2.5 eq. Cs_2CO_3	Na_2SO_4	-	Trace product
15	2.5 eq. NaOH	$MgSO_4$	-	90% yield[c]
15 x 2	2.5 eq. K_2CO_3	$MgSO_4$	15 minutes of rest at 25 °C in between the milling	Reaction incomplete after 1st mill, complete after 2nd milling
15 x 2	2.5 eq. K_2CO_3	$MgSO_4$	15 minutes of rest at 50° C[b] in between the milling	Reaction incomplete after 1st mill, complete after 2nd milling

[a]100 mg (0.48mmol) of **2**, 2 eq. of 3-pentanone, 600 mg milling auxiliary. 15 mL stainless steel jar, 10 mm stainless steel ball. The jars were sealed with paraffin.
[b]50 °C Oven.
[c]Further scaling up to 1 g scale in a 30 mL jar and 15 mm stainless steel ball produced similar results.

Supplementary Table 2. Condition scans for Diels-Alder reaction of 3 to yield 4.

Reaction conditions[a]				
**Milling time (h)**	**Dienophile[b]**	**Temperature[c] (°C)**	**Jar material[d]**	**Results/Observation**
**2**	NBD	35	SS	No **4** generated
**2**	NBD[e]	35	Zr	Trace amounts of **4** generated
**3**	DMAD[e]	35	SS	70% yield (**3b**)
**3**	DMAD	35	Zr	70% yield (**3b**)
**1**	DMAD	40[f]	SS	70% yield (**3b**)
**2**	NBD	45[f]	WC	Trace amounts of **4** generated
**2**	NBD	40[f]	SS	Trace amounts of **4** generated
**2**	NBD	95	SS	95% yield (**4**)

[a]100 mg of **3**, 600 mg $MgSO_4$, 15mL jar, 30 Hz. Ac_2O and TMBzACl were added as additives in 2 equivalents.
[b]8 equivalents of norbornadiene, 2 equivalents for dimethylacetylene carboxylate.
[c]Measured during milling, attained after 5 to 10 minutes of milling
[d]SS: Stainless Steel, Zr: Zirconia
[e]NBD: Norbornadiene, DMAD: Dimethylacetylene dicarboxylate
[f]2 10 mm stainless steel balls were used.

Supplementary Table 3. Solution phase benzylic bromination of 4.

Eq. of NBS	Eq. of AIBN	Volume of EtOAc (mL)	Conditions	Time (h)	Results/Observation
4.5	-	3	Ambient	3	Mixture of Di- and mono-brominated products
4.5	0.2	3	Ambient	3	78%
4.5	0.2	3	300W lamp	3	Complex product mixture
4.5	0.2	1	Ambient	3	Complex product mixture
4.5	0.2	9	Ambient	3	78%
9	-	3	Ambient	16	Mixture of di- and mono-brominated products
9	0.2	3	Ambient	16	Complex product mixture

[a]100 mg (0.39mmol) of **4**, refluxed in EtOAc

Supplementary Table 4. Mechanochemical ring closing

Precursor	Base	Salt	Milling Auxiliary	5-min Pre-milling	Results/Observation
**1**	NaOH	-	NaCl	No	2%
**1**	Na^{t}BuO	-	NaCl	No	5%
**1**	NaOH	TBACl	NaCl	Yes	32%
**1**	NaOH	TMBZCl	Na_2SO_4	Yes	27%
**1**	$Ba(OH)_2$	TBACl	Na_2SO_4	Yes	66%
**1**	$Ba(OH)_2$	TBACl	Na_2SO_4	No	57%[a]
**1**	$Ba(OH)_2$	TBACl	Na_2SO_4	No	64%[b]
**1**	$Ba(OH)_2$	TBACl	Na_2SO_4	No	60%[c]
**1**	$Ba(OH)_2$	TBABr	Na_2SO_4	Yes	5%
**1**	$Ba(OH)_2$	TBAF	Na_2SO_4	Yes	No product
**1**	-	TBACl	Na_2SO_4	Yes	No product
**8**	$Ba(OH)_2$	TBACl	Na_2SO_4	No	67%
**8**	$Ba(OH)_2$	-	Na_2SO_4	No	No product

[a]Milling time = 5 min, [b]Milling time = 10 min, [c]Milling time = 20 min

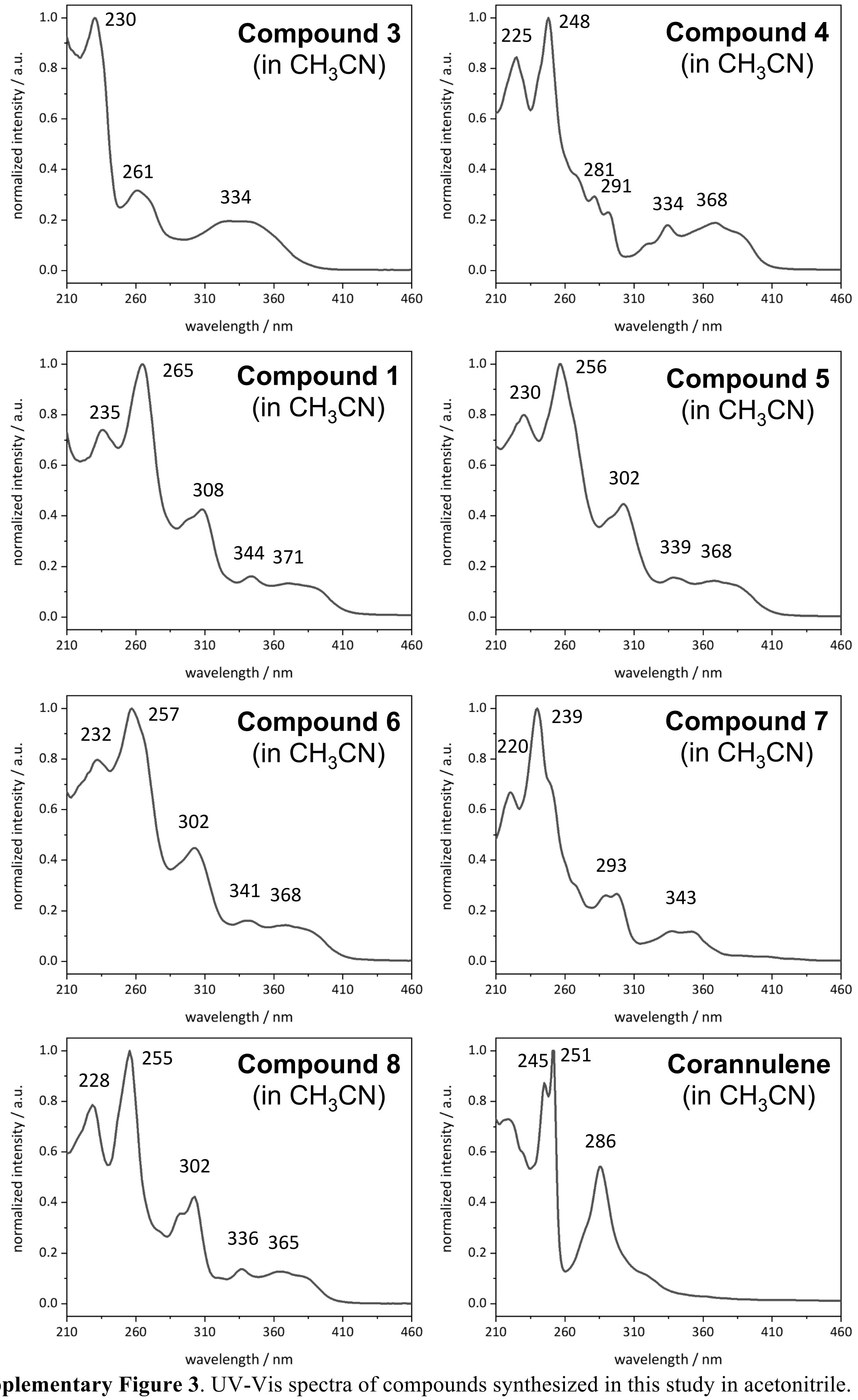

Supplementary Figure 3. UV-Vis spectra of compounds synthesized in this study in acetonitrile.

Compound 5

checkCIF/PLATON report

Structure factors have been supplied for datablock(s) ms76s

THIS REPORT IS FOR GUIDANCE ONLY. IF USED AS PART OF A REVIEW PROCEDURE FOR PUBLICATION, IT SHOULD NOT REPLACE THE EXPERTISE OF AN EXPERIENCED CRYSTALLOGRAPHIC REFEREE.

No syntax errors found. CIF dictionary Interpreting this report

Datablock: ms76s

```
Bond precision:   C-C = 0.0060 A          Wavelength=1.54178

Cell:             a=10.0857(5)     b=17.0532(10)      c=10.3993(5)
                  alpha=90         beta=93.353(3)     gamma=90
Temperature:      100 K

                 Calculated                 Reported
Volume           1785.55(16)                1785.55(16)
Space group      C 2/c                      C 1 2/c 1
Hall group       -C 2yc                     -C 2yc
Moiety formula   C20 H14 Br2.12 Cl1.88      ?
Sum formula      C20 H14 Br2.12 Cl1.88      C20 H14 Br2.12 Cl1.88
Mr               490.35                     490.37
Dx,g cm-3        1.824                      1.824
Z                4                          4
Mu (mm-1)        8.637                      8.637
F000             960.6                      961.0
F000'            959.12
h,k,lmax         12,20,12                   12,20,12
Nref             1629                       1608
Tmin,Tmax        0.694,0.841                0.230,0.850
Tmin'            0.085

Correction method= # Reported T Limits: Tmin=0.230 Tmax=0.850
AbsCorr = MULTI-SCAN

Data completeness= 0.987          Theta(max)= 68.080

R(reflections)= 0.0466( 1511)     wR2(reflections)= 0.1331( 1608)

S = 1.152                   Npar= 141
```

The following ALERTS were generated. Each ALERT has the format
test-name_ALERT_alert-type_alert-level.
Click on the hyperlinks for more details of the test.

Alert level C

```
PLAT077_ALERT_4_C Unitcell Contains Non-integer Number of Atoms ..     Please Check
PLAT250_ALERT_2_C Large U3/U1 Ratio for Average U(i,j) Tensor ....       2.1 Note
PLAT336_ALERT_2_C Long Bond Distance for ..... C1       -Cl1           1.998 Ang.
PLAT336_ALERT_2_C Long Bond Distance for ..... C11      -Cl2           1.999 Ang.
PLAT336_ALERT_2_C Long Bond Distance for ..... C11      -Cl3           1.997 Ang.
PLAT911_ALERT_3_C Missing FCF Refl Between Thmin & STh/L=    0.600        16 Report
```

Alert level G

```
PLAT002_ALERT_2_G Number of Distance or Angle Restraints on AtSite         7 Note
PLAT003_ALERT_2_G Number of Uiso or Uij Restrained non-H Atoms ...         5 Report
PLAT068_ALERT_1_G Reported F000 Differs from Calcd (or Missing)...     Please Check
PLAT176_ALERT_4_G The CIF-Embedded .res File Contains SADI Records         2 Report
PLAT178_ALERT_4_G The CIF-Embedded .res File Contains SIMU Records         1 Report
PLAT186_ALERT_4_G The CIF-Embedded .res File Contains ISOR Records         1 Report
PLAT301_ALERT_3_G Main Residue  Disorder ..............(Resd  1  )       17% Note
PLAT410_ALERT_2_G Short Intra H...H Contact  H1A      ..H11A     .      2.11 Ang.
                                                        x,y,z  =      1_555 Check
PLAT410_ALERT_2_G Short Intra H...H Contact  H1A      ..H11F     .      2.06 Ang.
                                                        x,y,z  =      1_555 Check
PLAT860_ALERT_3_G Number of Least-Squares Restraints .............        47 Note
PLAT909_ALERT_3_G Percentage of I>2sig(I) Data at Theta(Max) Still       84% Note
PLAT912_ALERT_4_G Missing # of FCF Reflections Above STh/L=  0.600         5 Note
PLAT941_ALERT_3_G Average HKL Measurement Multiplicity ...........       3.6 Low
PLAT978_ALERT_2_G Number C-C Bonds with Positive Residual Density.         0 Info
```

```
  0 ALERT level A = Most likely a serious problem - resolve or explain
  0 ALERT level B = A potentially serious problem, consider carefully
  6 ALERT level C = Check. Ensure it is not caused by an omission or oversight
 14 ALERT level G = General information/check it is not something unexpected

  1 ALERT type 1 CIF construction/syntax error, inconsistent or missing data
  9 ALERT type 2 Indicator that the structure model may be wrong or deficient
  5 ALERT type 3 Indicator that the structure quality may be low
  5 ALERT type 4 Improvement, methodology, query or suggestion
  0 ALERT type 5 Informative message, check
```

It is advisable to attempt to resolve as many as possible of the alerts in all categories. Often the minor alerts point to easily fixed oversights, errors and omissions in your CIF or refinement strategy, so attention to these fine details can be worthwhile. In order to resolve some of the more serious problems it may be necessary to carry out additional measurements or structure refinements. However, the purpose of your study may justify the reported deviations and the more serious of these should normally be commented upon in the discussion or experimental section of a paper or in the "special_details" fields of the CIF. checkCIF was carefully designed to identify outliers and unusual parameters, but every test has its limitations and alerts that are not important in a particular case may appear. Conversely, the absence of alerts does not guarantee there are no aspects of the results needing attention. It is up to the individual to critically assess their own results and, if necessary, seek expert advice.

Publication of your CIF in IUCr journals

A basic structural check has been run on your CIF. These basic checks will be run on all CIFs submitted for publication in IUCr journals (*Acta Crystallographica*, *Journal of Applied Crystallography*, *Journal of Synchrotron Radiation*); however, if you intend to submit to *Acta Crystallographica Section C* or *E* or *IUCrData*, you should make sure that full publication checks are run on the final version of your CIF prior to submission.

Publication of your CIF in other journals

Please refer to the *Notes for Authors* of the relevant journal for any special instructions relating to CIF submission.

PLATON version of 22/03/2021; check.def file version of 19/03/2021

Datablock ms76s - ellipsoid plot

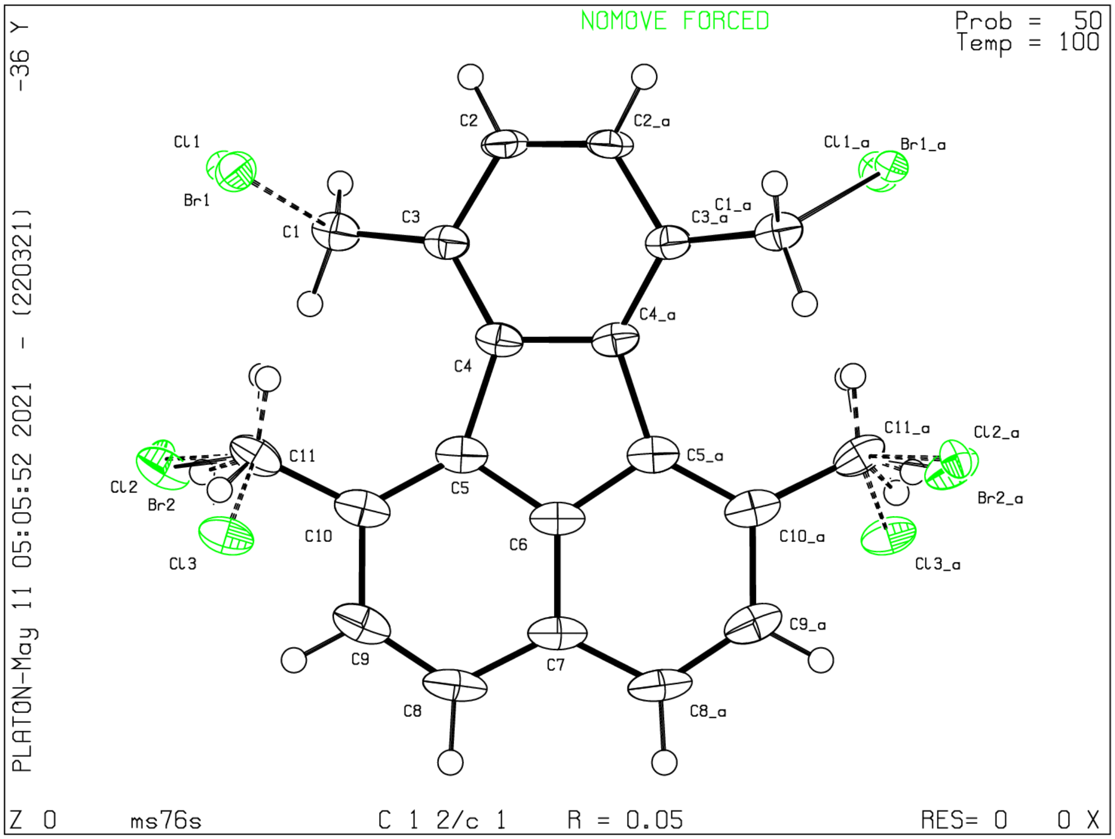

Compound 6

checkCIF/PLATON report

Structure factors have been supplied for datablock(s) ms71

THIS REPORT IS FOR GUIDANCE ONLY. IF USED AS PART OF A REVIEW PROCEDURE FOR PUBLICATION, IT SHOULD NOT REPLACE THE EXPERTISE OF AN EXPERIENCED CRYSTALLOGRAPHIC REFEREE.

No syntax errors found. CIF dictionary Interpreting this report

Datablock: ms71

```
Bond precision:   C-C = 0.0036 A          Wavelength=0.71073

Cell:            a=10.0760(14)    b=17.102(2)       c=10.4260(13)
                 alpha=90         beta=93.828(8)    gamma=90
Temperature:     100 K

                 Calculated                Reported
Volume           1792.6(4)                 1792.6(4)
Space group      C 2/c                     C 1 2/c 1
Hall group       -C 2yc                    -C 2yc
Moiety formula   C20 H14 Br3 Cl            ?
Sum formula      C20 H14 Br3 Cl            C20 H14 Br3 Cl
Mr               529.46                    529.49
Dx,g cm-3        1.962                     1.962
Z                4                         4
Mu (mm-1)        6.899                     6.899
F000             1024.0                    1024.0
F000'            1021.68
h,k,lmax         15,26,16                  15,26,16
Nref             3611                      3602
Tmin,Tmax        0.208,0.289               0.240,0.370
Tmin'            0.175

Correction method= # Reported T Limits: Tmin=0.240 Tmax=0.370
AbsCorr = MULTI-SCAN

Data completeness= 0.998          Theta(max)= 33.780

R(reflections)= 0.0412( 2404)     wR2(reflections)= 0.0903( 3602)

S = 1.002                  Npar= 128
```

The following ALERTS were generated. Each ALERT has the format
test-name_ALERT_alert-type_alert-level.
Click on the hyperlinks for more details of the test.

Alert level G

```
PLAT002_ALERT_2_G Number of Distance or Angle Restraints on AtSite          6 Note
PLAT003_ALERT_2_G Number of Uiso or Uij Restrained non-H Atoms ...          4 Report
PLAT176_ALERT_4_G The CIF-Embedded .res File Contains SADI Records          2 Report
PLAT178_ALERT_4_G The CIF-Embedded .res File Contains SIMU Records          1 Report
PLAT300_ALERT_4_G Atom Site Occupancy of Br1        Constrained at       0.75 Check
PLAT300_ALERT_4_G Atom Site Occupancy of Br2        Constrained at       0.75 Check
PLAT300_ALERT_4_G Atom Site Occupancy of Cl1        Constrained at       0.25 Check
PLAT300_ALERT_4_G Atom Site Occupancy of Cl2        Constrained at       0.25 Check
PLAT301_ALERT_3_G Main Residue  Disorder .............(Resd  1  )        17% Note
PLAT802_ALERT_4_G CIF Input Record(s) with more than 80 Characters          2 Info
PLAT860_ALERT_3_G Number of Least-Squares Restraints ............          14 Note
PLAT883_ALERT_1_G No Info/Value for _atom_sites_solution_primary .     Please Do !
PLAT912_ALERT_4_G Missing # of FCF Reflections Above STh/L=  0.600          8 Note
PLAT941_ALERT_3_G Average HKL Measurement Multiplicity ..........         3.4 Low
PLAT978_ALERT_2_G Number C-C Bonds with Positive Residual Density.          6 Info
```

```
  0 ALERT level A = Most likely a serious problem - resolve or explain
  0 ALERT level B = A potentially serious problem, consider carefully
  0 ALERT level C = Check. Ensure it is not caused by an omission or oversight
 15 ALERT level G = General information/check it is not something unexpected

  1 ALERT type 1 CIF construction/syntax error, inconsistent or missing data
  3 ALERT type 2 Indicator that the structure model may be wrong or deficient
  3 ALERT type 3 Indicator that the structure quality may be low
  8 ALERT type 4 Improvement, methodology, query or suggestion
  0 ALERT type 5 Informative message, check
```

It is advisable to attempt to resolve as many as possible of the alerts in all categories. Often the minor alerts point to easily fixed oversights, errors and omissions in your CIF or refinement strategy, so attention to these fine details can be worthwhile. In order to resolve some of the more serious problems it may be necessary to carry out additional measurements or structure refinements. However, the purpose of your study may justify the reported deviations and the more serious of these should normally be commented upon in the discussion or experimental section of a paper or in the "special_details" fields of the CIF. checkCIF was carefully designed to identify outliers and unusual parameters, but every test has its limitations and alerts that are not important in a particular case may appear. Conversely, the absence of alerts does not guarantee there are no aspects of the results needing attention. It is up to the individual to critically assess their own results and, if necessary, seek expert advice.

Publication of your CIF in IUCr journals

A basic structural check has been run on your CIF. These basic checks will be run on all CIFs submitted for publication in IUCr journals (*Acta Crystallographica*, *Journal of Applied Crystallography*, *Journal of Synchrotron Radiation*); however, if you intend to submit to *Acta Crystallographica Section C* or *E* or *IUCrData*, you should make sure that full publication checks are run on the final version of your CIF prior to submission.

Publication of your CIF in other journals

Please refer to the *Notes for Authors* of the relevant journal for any special instructions relating to CIF submission.

PLATON version of 22/03/2021; check.def file version of 19/03/2021

Datablock ms71 - ellipsoid plot

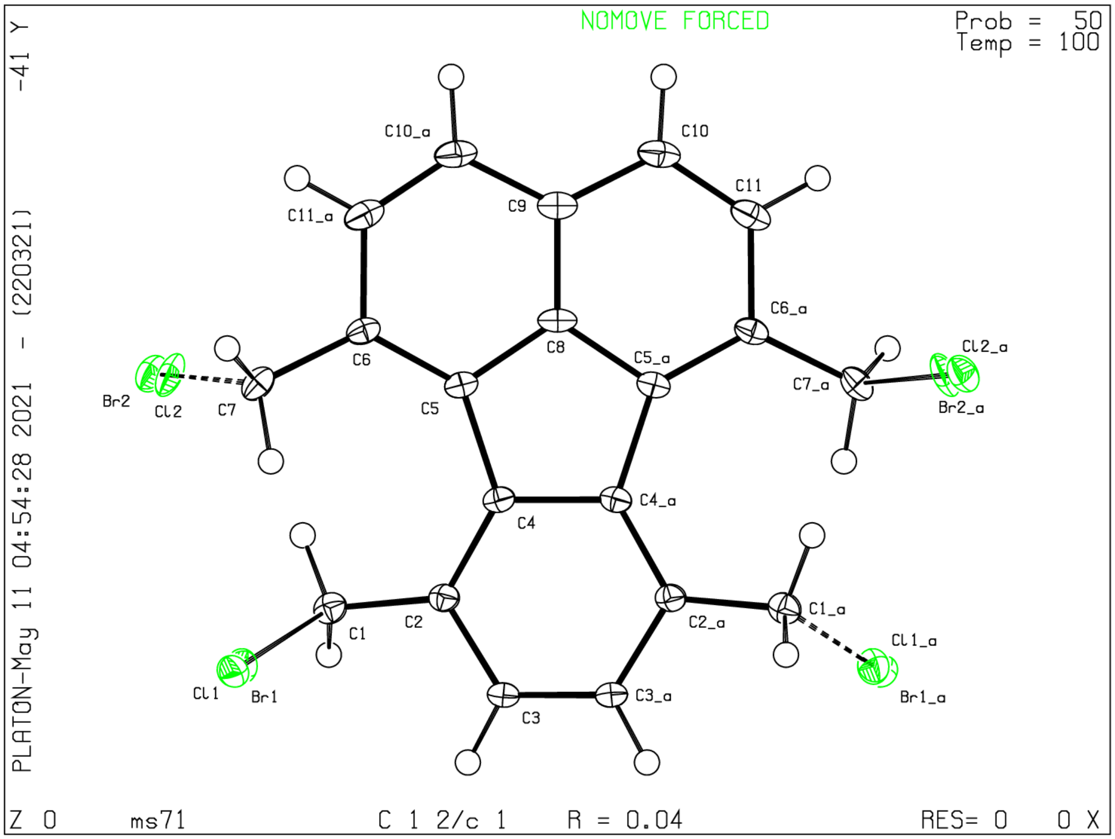

Compound 7

checkCIF/PLATON report

Structure factors have been supplied for datablock(s) ms72

THIS REPORT IS FOR GUIDANCE ONLY. IF USED AS PART OF A REVIEW PROCEDURE FOR PUBLICATION, IT SHOULD NOT REPLACE THE EXPERTISE OF AN EXPERIENCED CRYSTALLOGRAPHIC REFEREE.

No syntax errors found. CIF dictionary Interpreting this report

Datablock: ms72

```
Bond precision:   C-C = 0.0056 A          Wavelength=0.71073

Cell:              a=7.6775(10)       b=18.324(2)      c=20.208(3)
                   alpha=90           beta=90          gamma=90
Temperature:       100 K
```

	Calculated	Reported
Volume	2842.9(6)	2842.9(6)
Space group	P b c a	P b c a
Hall group	-P 2ac 2ab	-P 2ac 2ab
Moiety formula	C20 H12 Cl2	?
Sum formula	C20 H12 Cl2	C20 H12 Cl2
Mr	323.20	323.20
Dx,g cm-3	1.510	1.510
Z	8	8
Mu (mm-1)	0.448	0.448
F000	1328.0	1328.0
F000'	1330.78	
h,k,lmax	9,21,24	9,21,24
Nref	2535	2524
Tmin,Tmax	0.938,0.996	0.740,1.000
Tmin'	0.882	

```
Correction method= # Reported T Limits: Tmin=0.740 Tmax=1.000
AbsCorr = MULTI-SCAN

Data completeness= 0.996           Theta(max)= 25.130

R(reflections)= 0.0551( 1417)      wR2(reflections)= 0.1363( 2524)

S = 0.991                    Npar= 199
```

The following ALERTS were generated. Each ALERT has the format
test-name_ALERT_alert-type_alert-level.
Click on the hyperlinks for more details of the test.

Alert level C

```
RINTA01_ALERT_3_C  The value of Rint is greater than 0.12
           Rint given   0.152
PLAT340_ALERT_3_C Low Bond Precision on  C-C Bonds ...............    0.00558 Ang.
PLAT911_ALERT_3_C Missing FCF Refl Between Thmin & STh/L=    0.598         12 Report
```

Alert level G

```
PLAT020_ALERT_3_G The Value of Rint is Greater Than 0.12 .........      0.152 Report
PLAT434_ALERT_2_G Short Inter HL..HL Contact Cl1      ..Cl2              3.38 Ang.
                                    -1/2+x,3/2-y,1-z  =      4_466 Check
PLAT802_ALERT_4_G CIF Input Record(s) with more than 80 Characters          2 Info
PLAT883_ALERT_1_G No Info/Value for _atom_sites_solution_primary .     Please Do !
PLAT909_ALERT_3_G Percentage of I>2sig(I) Data at Theta(Max) Still        32% Note
PLAT978_ALERT_2_G Number C-C Bonds with Positive Residual Density.          0 Info
```

```
   0 ALERT level A = Most likely a serious problem - resolve or explain
   0 ALERT level B = A potentially serious problem, consider carefully
   3 ALERT level C = Check. Ensure it is not caused by an omission or oversight
   6 ALERT level G = General information/check it is not something unexpected

   1 ALERT type 1 CIF construction/syntax error, inconsistent or missing data
   2 ALERT type 2 Indicator that the structure model may be wrong or deficient
   5 ALERT type 3 Indicator that the structure quality may be low
   1 ALERT type 4 Improvement, methodology, query or suggestion
   0 ALERT type 5 Informative message, check
```

It is advisable to attempt to resolve as many as possible of the alerts in all categories. Often the minor alerts point to easily fixed oversights, errors and omissions in your CIF or refinement strategy, so attention to these fine details can be worthwhile. In order to resolve some of the more serious problems it may be necessary to carry out additional measurements or structure refinements. However, the purpose of your study may justify the reported deviations and the more serious of these should normally be commented upon in the discussion or experimental section of a paper or in the "special_details" fields of the CIF. checkCIF was carefully designed to identify outliers and unusual parameters, but every test has its limitations and alerts that are not important in a particular case may appear. Conversely, the absence of alerts does not guarantee there are no aspects of the results needing attention. It is up to the individual to critically assess their own results and, if necessary, seek expert advice.

Publication of your CIF in IUCr journals

A basic structural check has been run on your CIF. These basic checks will be run on all CIFs submitted for publication in IUCr journals (*Acta Crystallographica*, *Journal of Applied Crystallography*, *Journal of Synchrotron Radiation*); however, if you intend to submit to *Acta Crystallographica Section C* or *E* or *IUCrData*, you should make sure that full publication checks are run on the final version of your CIF prior to submission.

Publication of your CIF in other journals

Please refer to the *Notes for Authors* of the relevant journal for any special instructions relating to CIF submission.

PLATON version of 22/03/2021; check.def file version of 19/03/2021

Datablock ms72 - ellipsoid plot

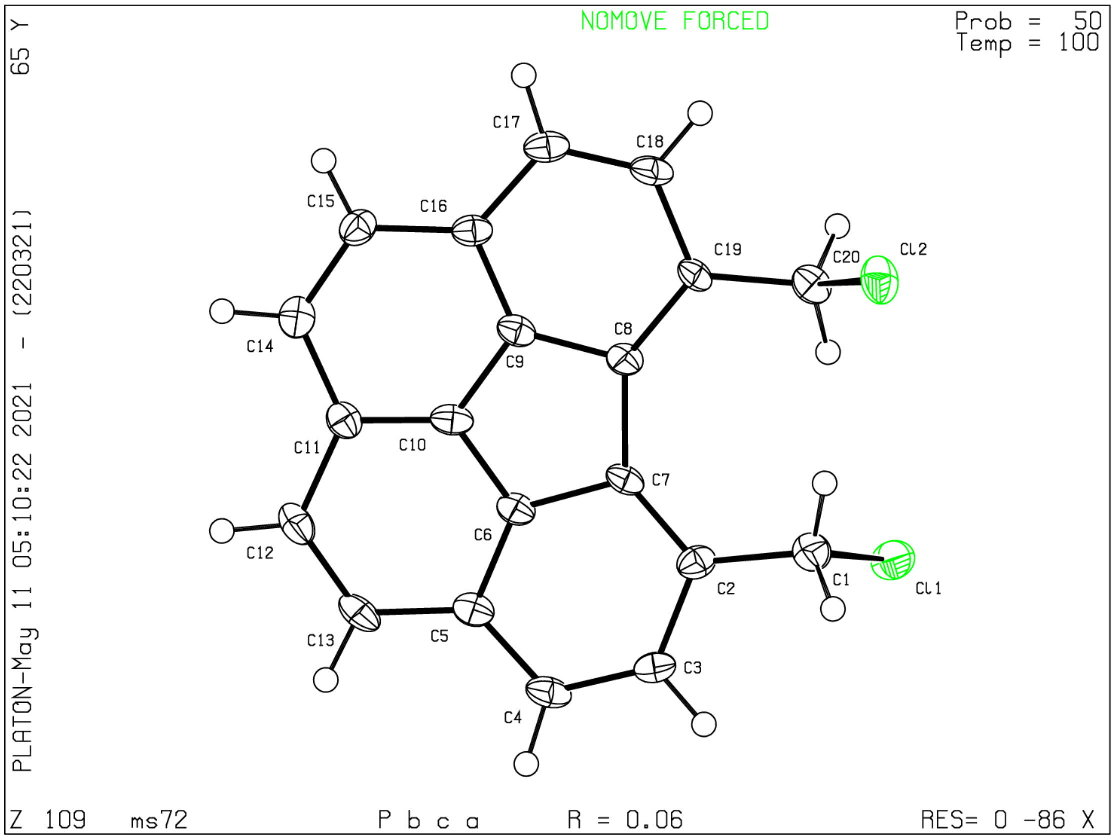

Compound 8

checkCIF/PLATON report

Structure factors have been supplied for datablock(s) ms83m

THIS REPORT IS FOR GUIDANCE ONLY. IF USED AS PART OF A REVIEW PROCEDURE FOR PUBLICATION, IT SHOULD NOT REPLACE THE EXPERTISE OF AN EXPERIENCED CRYSTALLOGRAPHIC REFEREE.

No syntax errors found. CIF dictionary Interpreting this report

Datablock: ms83m

```
Bond precision:   C-C = 0.0029 A          Wavelength=0.71073

Cell:             a=10.1095(6)     b=16.9176(10)        c=10.047(1)
                  alpha=90         beta=94.3453(16)     gamma=90
Temperature:      100 K

                  Calculated                  Reported
Volume            1713.4(2)                   1713.4(2)
Space group       C 2/c                       C 1 2/c 1
Hall group        -C 2yc                      -C 2yc
Moiety formula    C20 H14 Cl4                 ?
Sum formula       C20 H14 Cl4                 C20 H14 Cl4
Mr                396.11                      396.11
Dx,g cm-3         1.536                       1.536
Z                 4                           4
Mu (mm-1)         0.689                       0.689
F000              808.0                       808.0
F000'             810.58
h,k,lmax          14,23,14                    14,23,14
Nref              2514                        2514
Tmin,Tmax         0.984,0.993                 0.820,0.990
Tmin'             0.871

Correction method= # Reported T Limits: Tmin=0.820 Tmax=0.990
AbsCorr = MULTI-SCAN

Data completeness= 1.000          Theta(max)= 30.040

R(reflections)= 0.0392( 1923)     wR2(reflections)= 0.0999( 2514)

S = 1.015                    Npar= 110
```

The following ALERTS were generated. Each ALERT has the format
test-name_ALERT_alert-type_alert-level.
Click on the hyperlinks for more details of the test.

Alert level C

```
PLAT094_ALERT_2_C Ratio of Maximum / Minimum Residual Density ....       2.89 Report
```

Alert level G

```
PLAT802_ALERT_4_G CIF Input Record(s) with more than 80 Characters          2 Info
PLAT883_ALERT_1_G No Info/Value for _atom_sites_solution_primary .     Please Do !
PLAT913_ALERT_3_G Missing # of Very Strong Reflections in FCF ....          1 Note
PLAT978_ALERT_2_G Number C-C Bonds with Positive Residual Density.          3 Info
```

```
   0 ALERT level A = Most likely a serious problem - resolve or explain
   0 ALERT level B = A potentially serious problem, consider carefully
   1 ALERT level C = Check. Ensure it is not caused by an omission or oversight
   4 ALERT level G = General information/check it is not something unexpected

   1 ALERT type 1 CIF construction/syntax error, inconsistent or missing data
   2 ALERT type 2 Indicator that the structure model may be wrong or deficient
   1 ALERT type 3 Indicator that the structure quality may be low
   1 ALERT type 4 Improvement, methodology, query or suggestion
   0 ALERT type 5 Informative message, check
```

It is advisable to attempt to resolve as many as possible of the alerts in all categories. Often the minor alerts point to easily fixed oversights, errors and omissions in your CIF or refinement strategy, so attention to these fine details can be worthwhile. In order to resolve some of the more serious problems it may be necessary to carry out additional measurements or structure refinements. However, the purpose of your study may justify the reported deviations and the more serious of these should normally be commented upon in the discussion or experimental section of a paper or in the "special_details" fields of the CIF. checkCIF was carefully designed to identify outliers and unusual parameters, but every test has its limitations and alerts that are not important in a particular case may appear. Conversely, the absence of alerts does not guarantee there are no aspects of the results needing attention. It is up to the individual to critically assess their own results and, if necessary, seek expert advice.

Publication of your CIF in IUCr journals

A basic structural check has been run on your CIF. These basic checks will be run on all CIFs submitted for publication in IUCr journals (*Acta Crystallographica*, *Journal of Applied Crystallography*, *Journal of Synchrotron Radiation*); however, if you intend to submit to *Acta Crystallographica Section C* or *E* or *IUCrData*, you should make sure that full publication checks are run on the final version of your CIF prior to submission.

Publication of your CIF in other journals

Please refer to the *Notes for Authors* of the relevant journal for any special instructions relating to CIF submission.

PLATON version of 16/05/2021; check.def file version of 13/05/2021

Datablock ms83m - ellipsoid plot

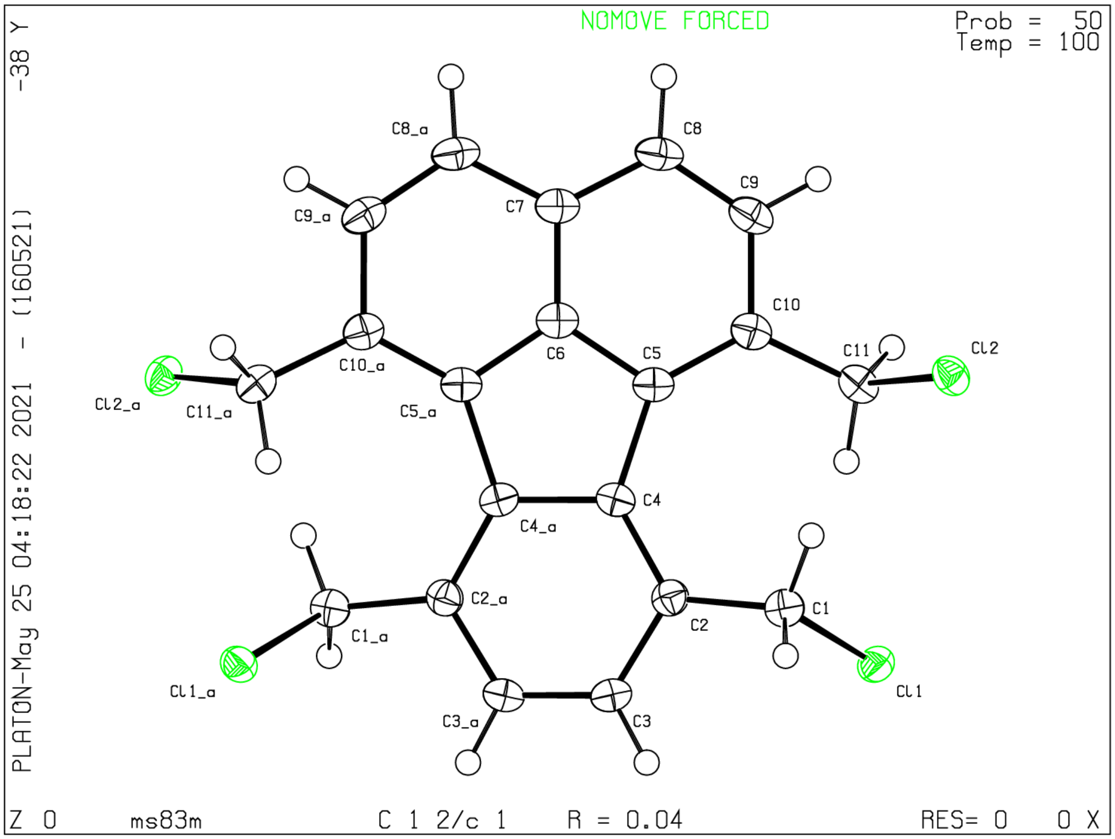

checkCIF/PLATON report

Structure factors have been supplied for datablock(s) ms73

THIS REPORT IS FOR GUIDANCE ONLY. IF USED AS PART OF A REVIEW PROCEDURE FOR PUBLICATION, IT SHOULD NOT REPLACE THE EXPERTISE OF AN EXPERIENCED CRYSTALLOGRAPHIC REFEREE.

No syntax errors found. CIF dictionary Interpreting this report

Datablock: ms73

```
Bond precision:   C-C = 0.0029 A          Wavelength=0.71073

Cell:             a=13.1202(6)     b=11.6149(7)       c=16.2292(10)
                  alpha=90         beta=102.178(4)    gamma=90
Temperature:      100 K

                  Calculated                Reported
Volume            2417.5(2)                 2417.5(2)
Space group       P 21/c                    P 1 21/c 1
Hall group        -P 2ybc                   -P 2ybc
Moiety formula    C20 H10                   ?
Sum formula       C20 H10                   C20 H10
Mr                250.28                    250.28
Dx,g cm-3         1.375                     1.375
Z                 8                         8
Mu (mm-1)         0.078                     0.078
F000              1040.0                    1040.0
F000'             1040.40
h,k,lmax          19,16,23                  19,16,23
Nref              7882                      7727
Tmin,Tmax         0.978,0.991               0.870,0.990
Tmin'             0.978

Correction method= # Reported T Limits: Tmin=0.870 Tmax=0.990
AbsCorr = MULTI-SCAN

Data completeness= 0.980          Theta(max)= 31.270

R(reflections)= 0.0652( 4638)     wR2(reflections)= 0.1547( 7727)

S = 1.014                   Npar= 361
```

The following ALERTS were generated. Each ALERT has the format
test-name_ALERT_alert-type_alert-level.
Click on the hyperlinks for more details of the test.

Alert level C

```
DIFMX02 ALERT 1 C  The maximum difference density is > 0.1*ZMAX*0.75
             The relevant atom site should be identified.
PLAT097 ALERT 2 C Large Reported Max.  (Positive) Residual Density       0.50 eA-3
PLAT906 ALERT 3 C Large K Value in the Analysis of Variance ......      4.911 Check
```

Alert level G

```
PLAT802 ALERT 4 G CIF Input Record(s) with more than 80 Characters         2 Info
PLAT883 ALERT 1 G No Info/Value for _atom_sites_solution_primary .     Please Do !
PLAT912 ALERT 4 G Missing # of FCF Reflections Above STh/L=  0.600       154 Note
PLAT941 ALERT 3 G Average HKL Measurement Multiplicity ..........        2.9 Low
PLAT978 ALERT 2 G Number C-C Bonds with Positive Residual Density.        17 Info
```

```
   0 ALERT level A = Most likely a serious problem - resolve or explain
   0 ALERT level B = A potentially serious problem, consider carefully
   3 ALERT level C = Check. Ensure it is not caused by an omission or oversight
   5 ALERT level G = General information/check it is not something unexpected

   2 ALERT type 1 CIF construction/syntax error, inconsistent or missing data
   2 ALERT type 2 Indicator that the structure model may be wrong or deficient
   2 ALERT type 3 Indicator that the structure quality may be low
   2 ALERT type 4 Improvement, methodology, query or suggestion
   0 ALERT type 5 Informative message, check
```

It is advisable to attempt to resolve as many as possible of the alerts in all categories. Often the minor alerts point to easily fixed oversights, errors and omissions in your CIF or refinement strategy, so attention to these fine details can be worthwhile. In order to resolve some of the more serious problems it may be necessary to carry out additional measurements or structure refinements. However, the purpose of your study may justify the reported deviations and the more serious of these should normally be commented upon in the discussion or experimental section of a paper or in the "special_details" fields of the CIF. checkCIF was carefully designed to identify outliers and unusual parameters, but every test has its limitations and alerts that are not important in a particular case may appear. Conversely, the absence of alerts does not guarantee there are no aspects of the results needing attention. It is up to the individual to critically assess their own results and, if necessary, seek expert advice.

Publication of your CIF in IUCr journals

A basic structural check has been run on your CIF. These basic checks will be run on all CIFs submitted for publication in IUCr journals (*Acta Crystallographica*, *Journal of Applied Crystallography*, *Journal of Synchrotron Radiation*); however, if you intend to submit to *Acta Crystallographica Section C* or *E* or *IUCrData*, you should make sure that full publication checks are run on the final version of your CIF prior to submission.

Publication of your CIF in other journals

Please refer to the *Notes for Authors* of the relevant journal for any special instructions relating to CIF submission.

PLATON version of 22/03/2021; check.def file version of 19/03/2021

Datablock ms73 - ellipsoid plot

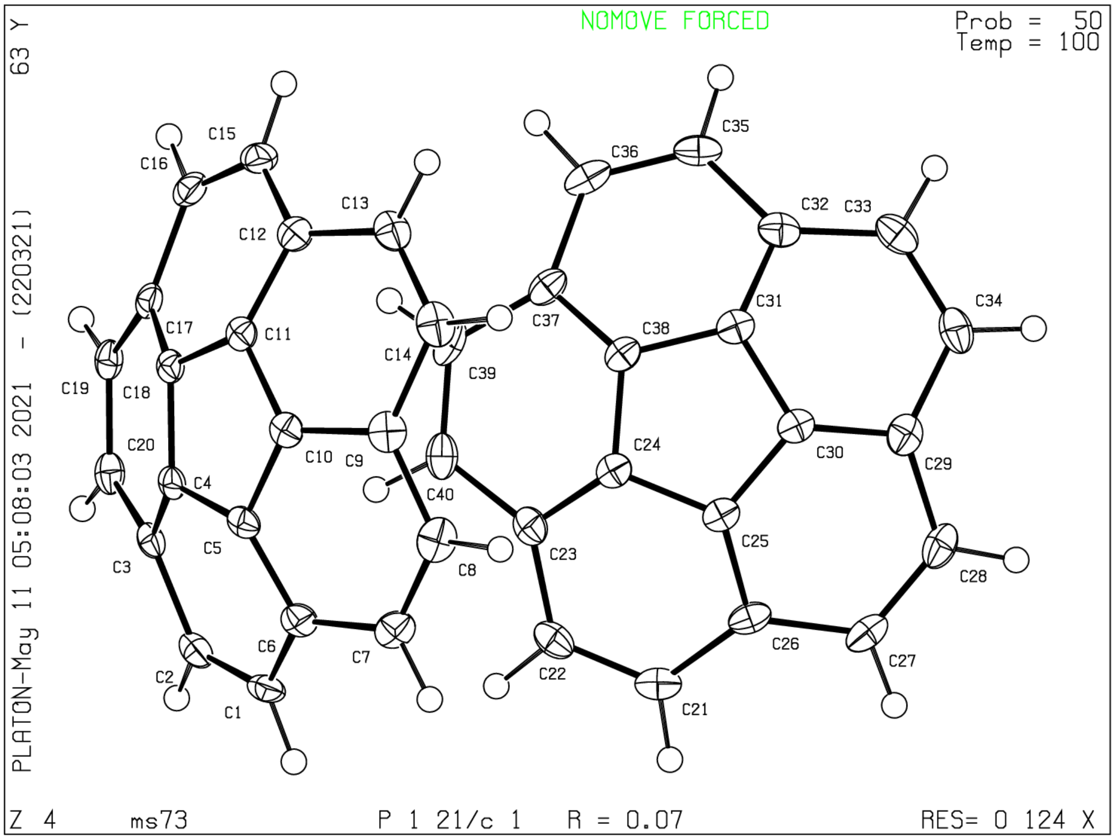

[Mass Spectrum]
Data : EI-A675   Date : 27-Apr-2021 14:04
RT : 0.47 min   Scan# : (44,64)
Elements : C 100/0, H 100/0, 79Br 5/0, 81Br 5/0
Mass Tolerance   : 10ppm, 5mmu if m/z < 500, 10mmu if m/z > 1000
Unsaturation (U.S.) : 0.0 - 20.0

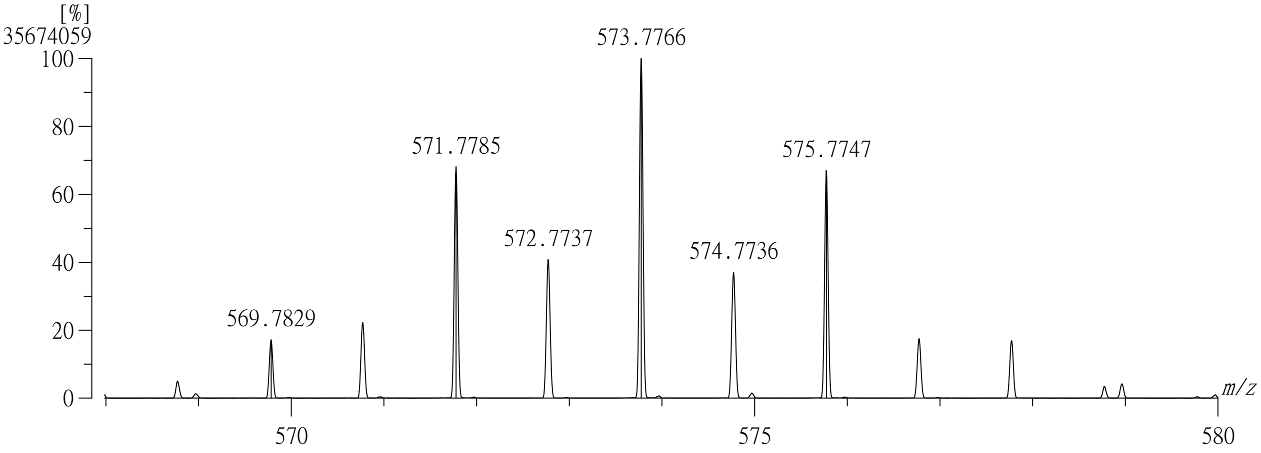

	Observed m/z	Int%	Err[ppm / mmu]	U.S.	Composition
1	569.7829	17.14	+0.0 / +0.0	12.0	C20 H14 79Br4
2	571.7785	68.10	-4.1 / -2.3	12.0	C20 H14 79Br3 81Br
3	573.7766	100.00	+8.6 / +4.9	0.0	C13 H23 79Br5
4			-3.8 / -2.2	12.0	C20 H14 79Br2 81Br2
5	575.7747	66.94	+8.8 / +5.1	0.0	C13 H23 79Br4 81Br
6			-3.6 / -2.1	12.0	C20 H14 79Br 81Br3

[Theoretical Ion Distribution]
Molecular Formula : C20 H14 Br4
(m/z 569.7829, MW 573.9472, U.S. 12.0)
Base Peak : 573.7789, Averaged MW : 573.9444(a), 573.9517(w)

```
  m/z      INT.
569.7829  17.3332**********
570.7862   3.8557**
571.7809  67.8525***************************************
572.7842  15.0301*********
573.7789 100.0000**********************************************************
574.7822  21.9976*************
575.7771  66.1409**************************************
576.7802  14.3517********
577.7755  17.0287**********
578.7783   3.5530**
579.7815   0.3696
580.7848   0.0245
581.7882   0.0012
```

Supplementary Figure 4. HRMS details of compound 1.

[Mass Spectrum]
Data : EI-A661   Date : 23-Apr-2021 14:17
RT : 0.24 min   Scan# : (6,15)
Elements : C 100/0, H 100/0, O 10/0
Mass Tolerance   : 20ppm, 5mmu if m/z < 250, 20mmu if m/z > 1000
Unsaturation (U.S.) : -0.5 - 30.0

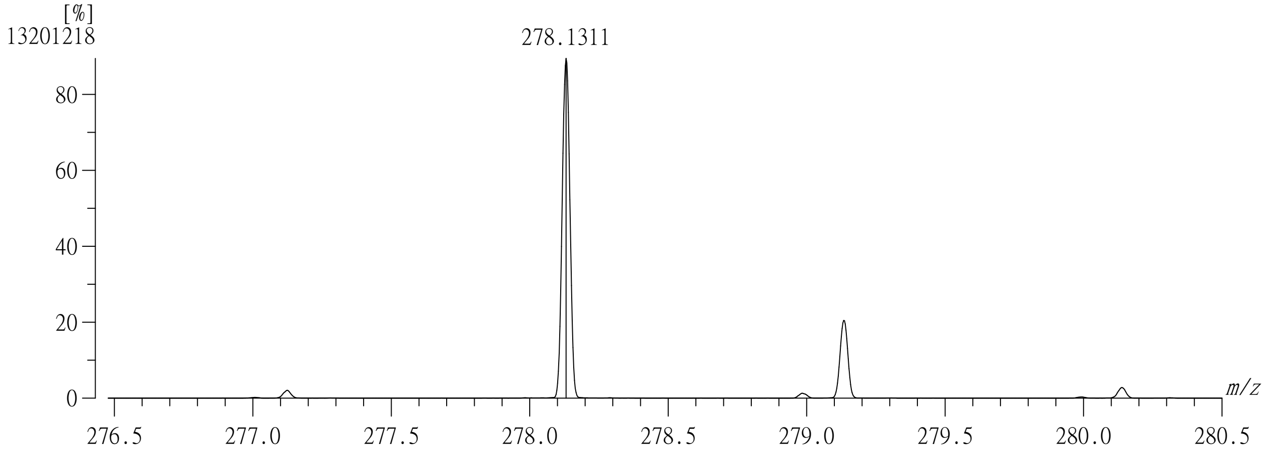

	Observed m/z	Int%	Err[ppm / mmu]	U.S.	Composition
1	278.1311	89.47	+1.5 / +0.4	11.0	C19 H18 O2
2			-19.6 / -5.5	2.0	C12 H22 O7

[Theoretical Ion Distribution]
Molecular Formula : C19 H18 O2
(m/z 278.1307, MW 278.3507, U.S. 11.0)
Base Peak : 278.1307, Averaged MW : 278.3492(a), 278.3500(w)

m/z	INT.
278.1307	100.0000**
279.1340	21.2086************
280.1370	2.5325*
281.1398	0.2198
282.1426	0.0149
283.1453	0.0008

Supplementary Figure 5. HRMS details of compound 3.

[Mass Spectrum]
Data : EI-A667 Date : 23-Apr-2021 16:21
RT : 0.23 min Scan# : (19,24)
Elements : C 100/0, H 100/0, O 10/0
Mass Tolerance : 10ppm, 5mmu if m/z < 500, 10mmu if m/z > 1000
Unsaturation (U.S.) : -0.5 - 20.0

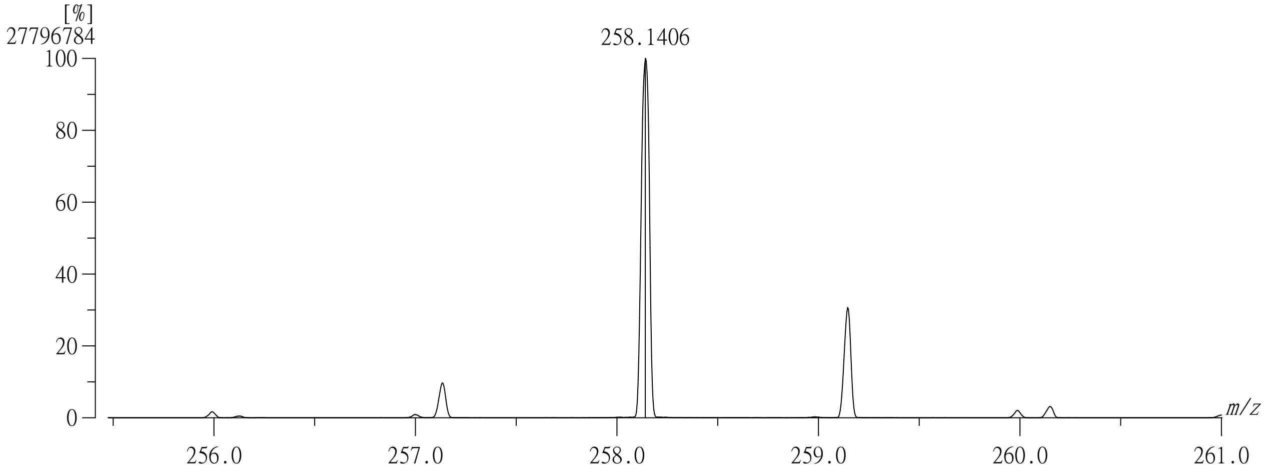

	Observed m/z	Int%	Err[ppm / mmu]	U.S.	Composition
1	258.1406	100.00	-1.0 / -0.3	12.0	C20 H18

[Theoretical Ion Distribution]
Molecular Formula : C20 H18
(m/z 258.1409, MW 258.3629, U.S. 12.0)
Base Peak : 258.1409, Averaged MW : 258.3616(a), 258.3624(w)

m/z	INT.
258.1409	100.0000**
259.1442	22.2447*************
260.1476	2.3504*
261.1509	0.1569
262.1543	0.0074
263.1576	0.0003

Supplementary Figure 6. HRMS details of compound 4.

[Mass Spectrum]
Data : EI-A671   Date : 27-Apr-2021 13:26
RT : 1.60 min   Scan# : (149,174)
Elements : C 100/0, H 100/0, 79Br 3/0, 81Br 3/0, 35Cl 3/0, 37Cl 3/0
Mass Tolerance   : 10ppm, 5mmu if m/z < 500, 10mmu if m/z > 1000
Unsaturation (U.S.) : 0.0 - 20.0

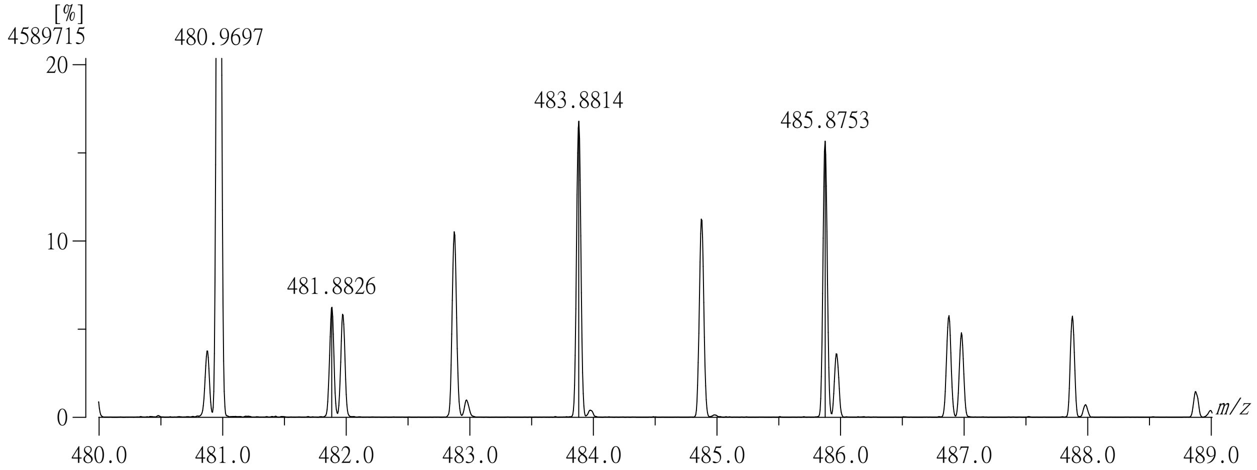

	Observed m/z	Int%	Err[ppm / mmu]	U.S.	Composition
1	481.8826	6.24	-2.8 / -1.3	12.0	C20 H14 79Br2 35Cl2
2			-5.9 / -2.8	19.0	C24 H8 81Br 35Cl3
3			-9.0 / -4.3	3.0	C15 H22 81Br3 37Cl
4			-4.0 / -1.9	19.0	C24 H8 79Br 35Cl2 37Cl
5			+1.4 / +0.7	5.0	C16 H19 81Br2 35Cl 37Cl2
6			+3.3 / +1.6	5.0	C16 H19 79Br 81Br 37Cl3
7	483.8814	16.79	-9.5 / -4.6	10.0	C19 H17 79Br2 81Br
8			+2.1 / +1.0	5.0	C16 H20 79Br3 35Cl
9			-1.0 / -0.5	12.0	C20 H14 79Br 81Br 35Cl2
10			+0.9 / +0.4	12.0	C20 H14 79Br2 35Cl 37Cl
11			-2.3 / -1.1	19.0	C24 H8 81Br 35Cl2 37Cl
12			+9.4 / +4.5	14.0	C21 H11 79Br 35Cl3 37Cl
13			-0.4 / -0.2	19.0	C24 H8 79Br 35Cl 37Cl2
14			+5.0 / +2.4	5.0	C16 H19 81Br2 37Cl3
15	485.8753	15.66	-6.2 / -3.0	5.0	C16 H20 79Br2 81Br 35Cl
16			+5.4 / +2.6	0.0	C13 H23 79Br3 35Cl2
17			-9.3 / -4.5	12.0	C20 H14 81Br2 35Cl2
18			+2.2 / +1.1	7.0	C17 H17 79Br 81Br 35Cl3
19			-4.3 / -2.1	5.0	C16 H20 79Br3 37Cl
20			-7.5 / -3.6	12.0	C20 H14 79Br 81Br 35Cl 37Cl
21			+4.1 / +2.0	7.0	C17 H17 79Br2 35Cl2 37Cl
22			+1.0 / +0.5	14.0	C21 H11 81Br 35Cl3 37Cl
23			-5.6 / -2.7	12.0	C20 H14 79Br2 37Cl2
24			-8.7 / -4.2	19.0	C24 H8 81Br 35Cl 37Cl2
25			+2.8 / +1.4	14.0	C21 H11 79Br 35Cl2 37Cl2
26			-6.9 / -3.3	19.0	C24 H8 79Br 37Cl3
27			+8.3 / +4.0	0.0	C13 H22 81Br2 35Cl 37Cl3

[Theoretical Ion Distribution]
Molecular Formula : C20 H14 Br2 Cl2
(m/z 481.8839, MW 485.0452, U.S. 12.0)
Base Peak : 483.8817, Averaged MW : 485.0428(a), 485.0504(w)

m/z	INT.
481.8839	38.3344***********************
482.8873	8.5274*****
483.8817	100.0000***
484.8850	22.1044*************
485.8795	90.2277***
486.8827	19.7076***********
487.8773	32.9007*******************
488.8802	6.9956****
489.8755	4.4407***
490.8778	0.8738*
491.8809	0.0895
492.8841	0.0059
493.8874	0.0003

Supplementary Figure 7. HRMS details of compound 5.

[Mass Spectrum]
Data : EI-A673   Date : 27-Apr-2021 13:51
RT : 1.83 min   Scan# : (206,235)
Elements : C 100/0, H 100/0, 79Br 4/0, 81Br 4/0, 35Cl 1/0, 37Cl 1/0
Mass Tolerance   : 10ppm, 5mmu if m/z < 500, 10mmu if m/z > 1000
Unsaturation (U.S.) : -0.5 - 20.0

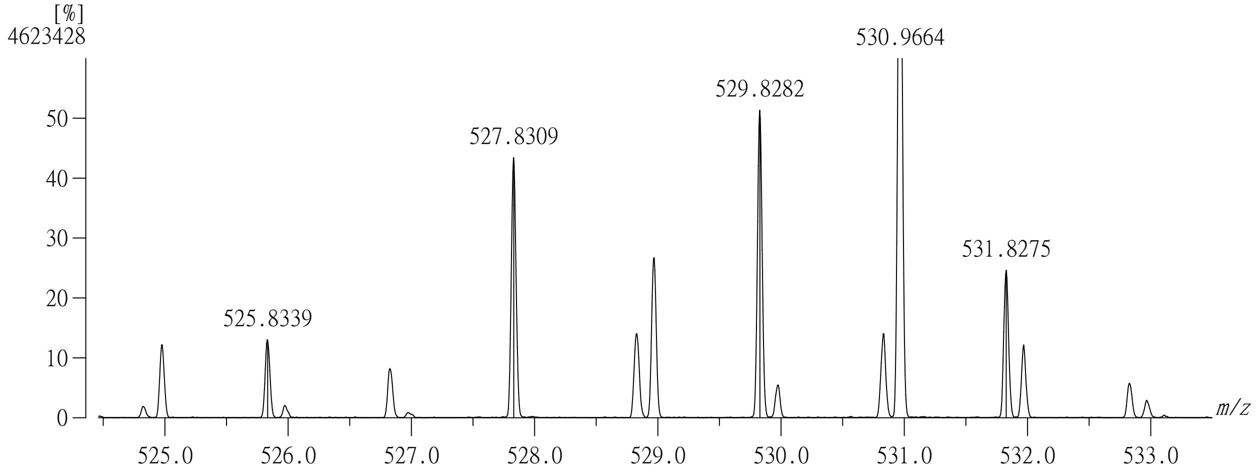

	Observed m/z	Int%	Err[ppm / mmu]	U.S.	Composition
1	525.8339	13.02	-9.8 / -5.1	17.0	C23 H11 79Br2 81Br
2			-6.5 / -3.4	3.0	C15 H22 81Br4
3			+0.9 / +0.5	12.0	C20 H14 79Br3 35Cl
4			-0.2 / -0.1	19.0	C24 H8 79Br2 35Cl 37Cl
5			+3.0 / +1.6	5.0	C16 H19 81Br3 35Cl 37Cl
6	527.8309	43.41	+2.0 / +1.1	5.0	C16 H20 79Br4
7			-0.9 / -0.5	12.0	C20 H14 79Br2 81Br 35Cl
8			+0.8 / +0.4	12.0	C20 H14 79Br3 37Cl
9			-2.0 / -1.1	19.0	C24 H8 79Br 81Br 35Cl 37Cl
10	529.8282	51.31	+0.8 / +0.4	5.0	C16 H20 79Br3 81Br
11			-2.1 / -1.1	12.0	C20 H14 79Br 81Br2 35Cl
12			-0.4 / -0.2	12.0	C20 H14 79Br2 81Br 37Cl
13			-3.3 / -1.7	19.0	C24 H8 81Br2 35Cl 37Cl
14	531.8275	24.61	+3.3 / +1.7	5.0	C16 H20 79Br2 81Br2
15			+0.4 / +0.2	12.0	C20 H14 81Br3 35Cl
16			+2.1 / +1.1	12.0	C20 H14 79Br 81Br2 37Cl

[Theoretical Ion Distribution]
Molecular Formula : C20 H14 Br3 Cl
(m/z 525.8334, MW 529.4962, U.S. 12.0)
Base Peak : 529.8293, Averaged MW : 529.4936(a), 529.5011(w)

m/z INT.
525.8334 25.9855***************
526.8368 5.7804***
527.8313 84.7548**
528.8347 18.7583***********
529.8293 100.0000***
530.8325 21.9364*************
531.8272 49.8210*****************************
532.8303 10.7226******
533.8255 8.7734*****
534.8281 1.7764*
535.8312 0.1833
536.8345 0.0121
537.8378 0.0006

Supplementary Figure 8. HRMS details of compound 6.

[Mass Spectrum]
Data : EI-A669 Date : 23-Apr-2021 16:41
RT : 1.06 min Scan# : (53,110)
Elements : C 100/0, H 100/0, 35Cl 3/0, 37Cl 3/0
Mass Tolerance : 10ppm, 5mmu if m/z < 500, 10mmu if m/z > 1000
Unsaturation (U.S.) : -0.5 - 20.0

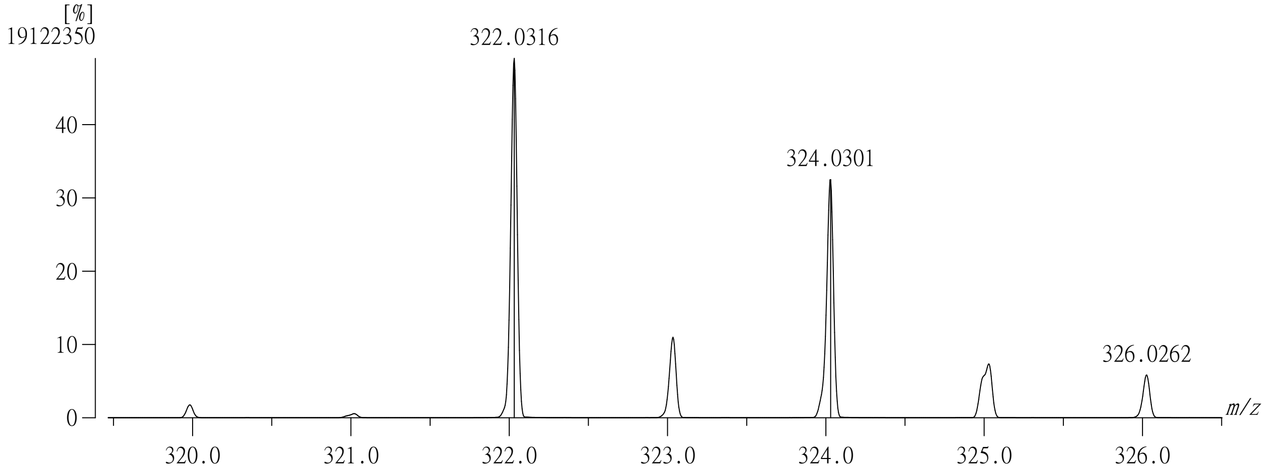

	Observed m/z	Int%	Err[ppm / mmu]	U.S.	Composition
1	322.0316	49.03	-0.0 / -0.0	14.0	C20 H12 35Cl2
2			-14.7 / -4.7	19.0	C23 H9 37Cl
3	324.0301	32.48	+4.5 / +1.4	14.0	C20 H12 35Cl 37Cl

[Theoretical Ion Distribution]
Molecular Formula : C20 H12 Cl2
(m/z 322.0316, MW 323.2213, U.S. 14.0)
Base Peak : 322.0316, Averaged MW : 323.2201(a), 323.2253(w)

m/z	INT.
322.0316	100.0000**
323.0350	22.2447*************
324.0290	66.3071***************************************
325.0321	14.3838********
326.0270	11.7368*******
327.0295	2.3754*
328.0326	0.2451
329.0359	0.0162
330.0392	0.0008

Supplementary Figure 9. HRMS details of compound 7.

[Mass Spectrum]
Data : EI-A773 Date : 28-May-2021 17:01
RT : 1.44 min Scan# : (86,90)
Elements : C 100/0, H 100/0, 35Cl 5/0, 37Cl 5/0
Mass Tolerance : 1000ppm, 5mmu if m/z < 5, 50mmu if m/z > 50
Unsaturation (U.S.) : -0.5 - 20.0

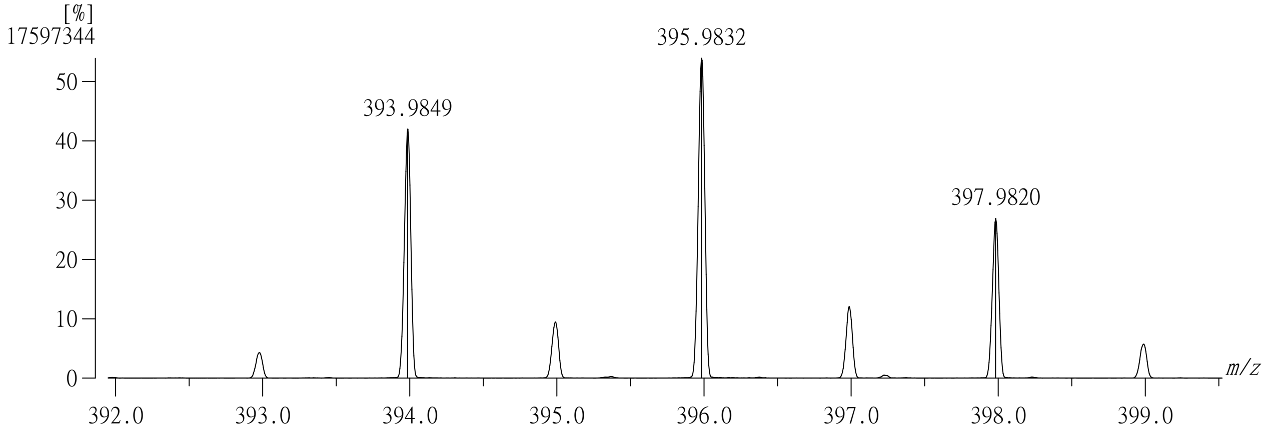

	Observed m/z	Int%	Err[ppm / mmu]	U.S.	Composition
1	393.9849	41.97	-118.5 / -46.7	20.0	C26 H12 35Cl2
2			-59.4 / -23.4	16.0	C23 H13 35Cl3
3			-0.2 / -0.1	12.0	C20 H14 35Cl4
4			+59.0 / +23.3	8.0	C17 H15 35Cl5
5			-12.1 / -4.8	17.0	C23 H11 35Cl2 37Cl
6			+47.1 / +18.5	13.0	C20 H12 35Cl3 37Cl
7			+106.3 / +41.9	9.0	C17 H13 35Cl4 37Cl
8			+35.1 / +13.8	18.0	C23 H9 35Cl 37Cl2
9			+94.3 / +37.1	14.0	C20 H10 35Cl2 37Cl2
10			-84.9 / -33.4	3.0	C16 H23 35Cl3 37Cl2
11			+82.3 / +32.4	19.0	C23 H7 37Cl3
12			-96.9 / -38.2	8.0	C19 H20 35Cl 37Cl3
13			-37.7 / -14.8	4.0	C16 H21 35Cl2 37Cl3
14			+21.5 / +8.5	0.0	C13 H22 35Cl3 37Cl3
15			-49.6 / -19.6	9.0	C19 H18 37Cl4
16			+9.5 / +3.8	5.0	C16 H19 35Cl 37Cl4
17			+68.7 / +27.1	1.0	C13 H20 35Cl2 37Cl4
18			+56.8 / +22.4	6.0	C16 H17 37Cl5
19			+116.0 / +45.7	2.0	C13 H18 35Cl 37Cl5
20	395.9832	53.90	-102.9 / -40.7	15.0	C23 H15 35Cl3
21			-44.0 / -17.4	11.0	C20 H16 35Cl4
22			+14.9 / +5.9	7.0	C17 H17 35Cl5
23			-114.8 / -45.5	20.0	C26 H12 35Cl 37Cl
24			-55.9 / -22.1	16.0	C23 H13 35Cl2 37Cl
25			+3.0 / +1.2	12.0	C20 H14 35Cl3 37Cl
26			+61.9 / +24.5	8.0	C17 H15 35Cl4 37Cl
27			+120.8 / +47.8	4.0	C14 H16 35Cl5 37Cl
28			-8.9 / -3.5	17.0	C23 H11 35Cl 37Cl2
29			+50.0 / +19.8	13.0	C20 H12 35Cl2 37Cl2
30			+108.9 / +43.1	9.0	C17 H13 35Cl3 37Cl2
31			+38.0 / +15.1	18.0	C23 H9 37Cl3
32			+96.9 / +38.4	14.0	C20 H10 35Cl 37Cl3
33			-81.3 / -32.2	3.0	C16 H23 35Cl2 37Cl3
34			-93.2 / -36.9	8.0	C19 H20 37Cl4
35			-34.3 / -13.6	4.0	C16 H21 35Cl 37Cl4
36			+24.6 / +9.7	0.0	C13 H22 35Cl2 37Cl4
37			+12.7 / +5.0	5.0	C16 H19 37Cl5
38			+71.6 / +28.3	1.0	C13 H20 35Cl 37Cl5
39	397.9820	26.90	-86.1 / -34.3	10.0	C20 H18 35Cl4
40			-27.5 / -10.9	6.0	C17 H19 35Cl5
41			-98.0 / -39.0	15.0	C23 H15 35Cl2 37Cl
42			-39.4 / -15.7	11.0	C20 H16 35Cl3 37Cl
43			+19.2 / +7.7	7.0	C17 H17 35Cl4 37Cl
44			+77.9 / +31.0	3.0	C14 H18 35Cl5 37Cl
45			-109.8 / -43.7	20.0	C26 H12 37Cl2
46			-51.2 / -20.4	16.0	C23 H13 35Cl 37Cl2
47			+7.4 / +2.9	12.0	C20 H14 35Cl2 37Cl2
48			+66.0 / +26.3	8.0	C17 H15 35Cl3 37Cl2
49			+124.6 / +49.6	4.0	C14 H16 35Cl4 37Cl2
50			-4.5 / -1.8	17.0	C23 H11 37Cl3
51			+54.1 / +21.5	13.0	C20 H12 35Cl 37Cl3
52			+112.7 / +44.9	9.0	C17 H13 35Cl2 37Cl3

50	−4.5 / −1.8	17.0	C23 H11 37Cl3
51	+54.1 / +21.5	13.0	C20 H12 35Cl 37Cl3
52	+112.7 / +44.9	9.0	C17 H13 35Cl2 37Cl3
53	−123.2 / −49.0	2.0	C16 H25 35Cl2 37Cl3
54	+100.9 / +40.1	14.0	C20 H10 37Cl4
55	−76.5 / −30.4	3.0	C16 H23 35Cl 37Cl4
56	−29.7 / −11.8	4.0	C16 H21 37Cl5
57	+28.9 / +11.5	0.0	C13 H22 35Cl 37Cl5

[Theoretical Ion Distribution]
Molecular Formula : C20 H14 Cl4
(m/z 393.9850, MW 396.1432, U.S. 12.0)
Base Peak : 395.9822, Averaged MW : 396.1412(a), 396.1492(w)

m/z	INT.	
393.9850	76.7673	**
394.9883	17.0766	**********
395.9822	100.0000	**
396.9854	21.9637	*************
397.9795	49.4157	*****************************
398.9826	10.6319	******
399.9771	11.1560	******
400.9798	2.3079	*
401.9754	1.0423	*
402.9773	0.1945	
403.9802	0.0196	
404.9834	0.0013	

Supplementary Figure 10. HRMS details of compound 8.

[Mass Spectrum]
Data : EI-A668 Date : 23-Apr-2021 16:31
RT : 0.22 min Scan# : (18,66)
Elements : C 100/0, H 100/0, O 10/0
Mass Tolerance : 10ppm, 5mmu if m/z < 500, 10mmu if m/z > 1000
Unsaturation (U.S.) : -0.5 - 30.0

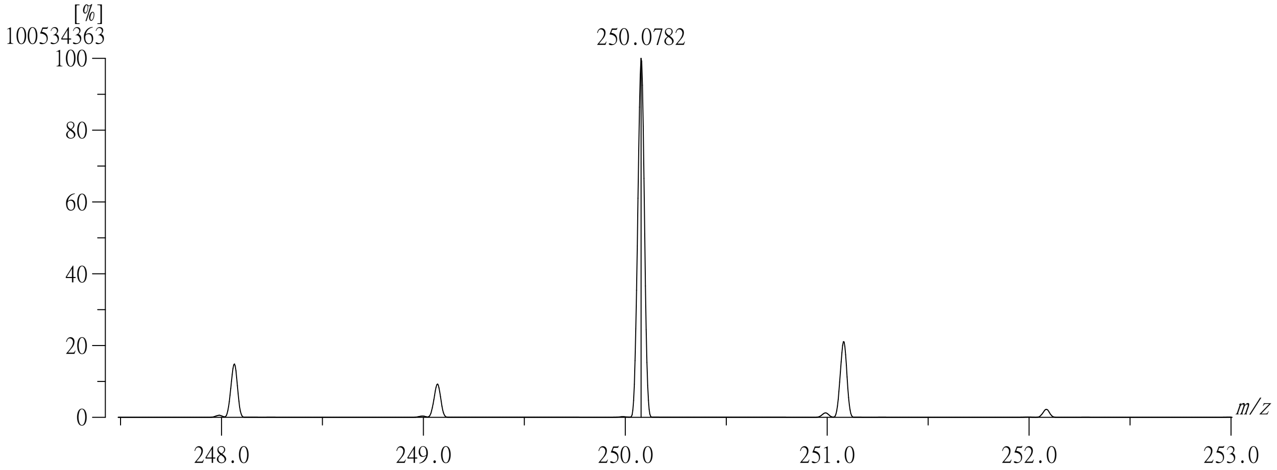

	Observed m/z	Int%	Err[ppm / mmu]	U.S.	Composition
1	250.0782	100.00	-0.2 / -0.1	16.0	C20 H10

[Theoretical Ion Distribution]
Molecular Formula : C20 H10
(m/z 250.0783, MW 250.2994, U.S. 16.0)
Base Peak : 250.0783, Averaged MW : 250.2990(a), 250.2999(w)

m/z	INT.	
250.0783	100.0000	**
251.0816	22.2447	*************
252.0850	2.3504	*
253.0883	0.1569	
254.0917	0.0074	
255.0950	0.0003	

Supplementary Figure 11. HRMS details of corannulene.

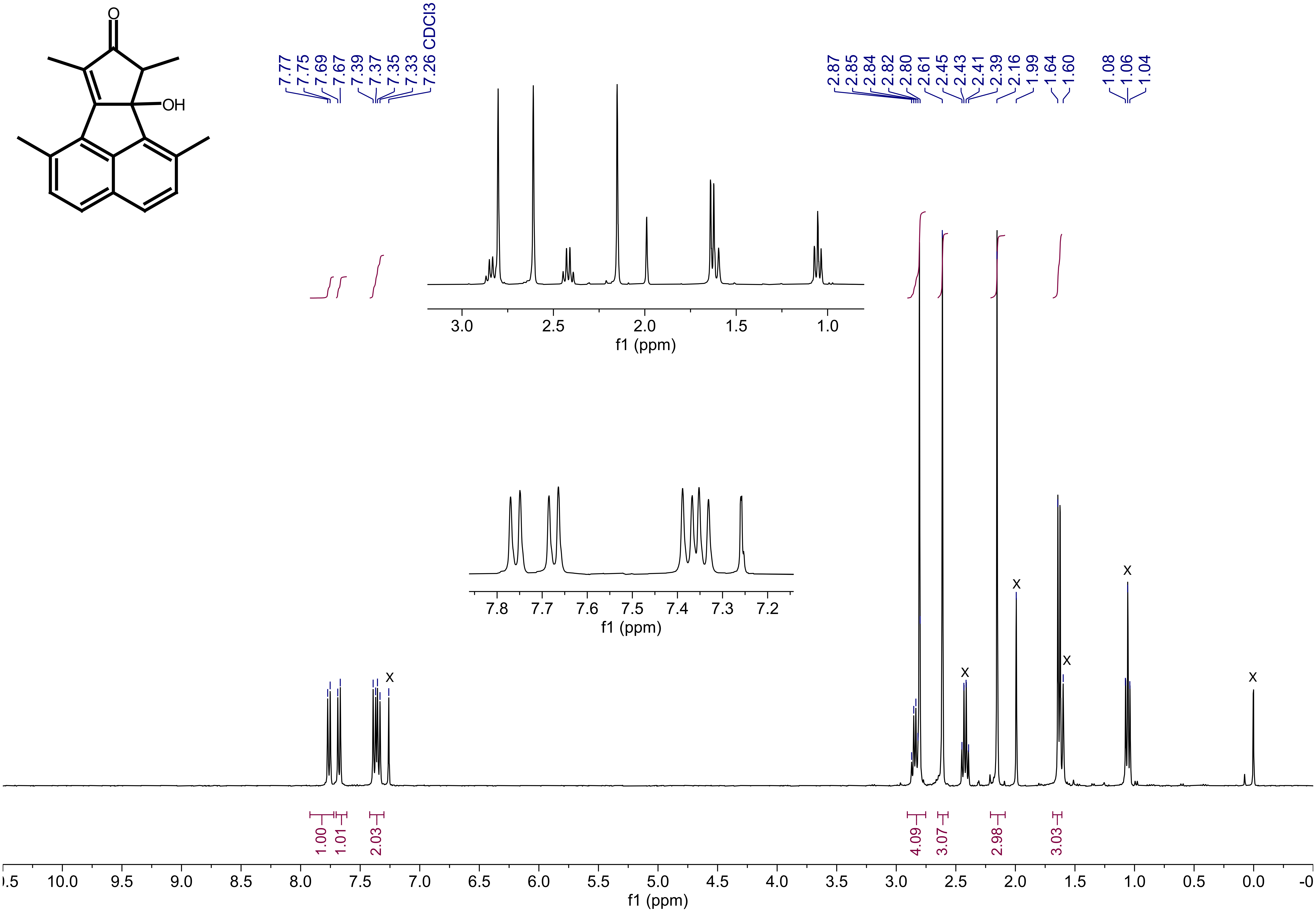

Supplementary Figure 12. Proton NMR of crude 3 in deuterated chloroform. Signals from TMS, 3-pentanone, acetone, water, and chloroform are shown with the help of a cross sign.

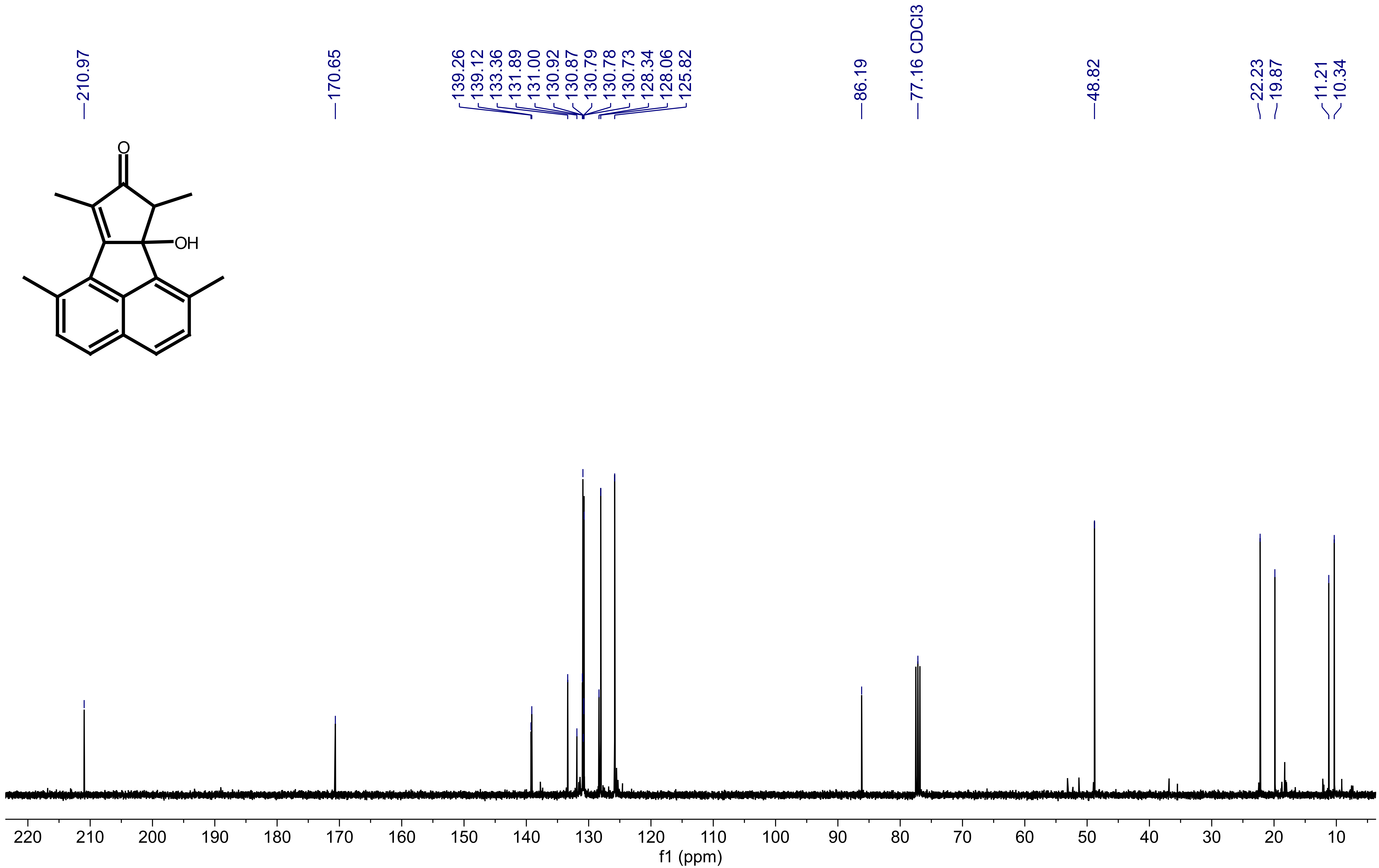

Supplementary Figure 13. Carbon-13 NMR of crude 3 in deuterated chloroform.

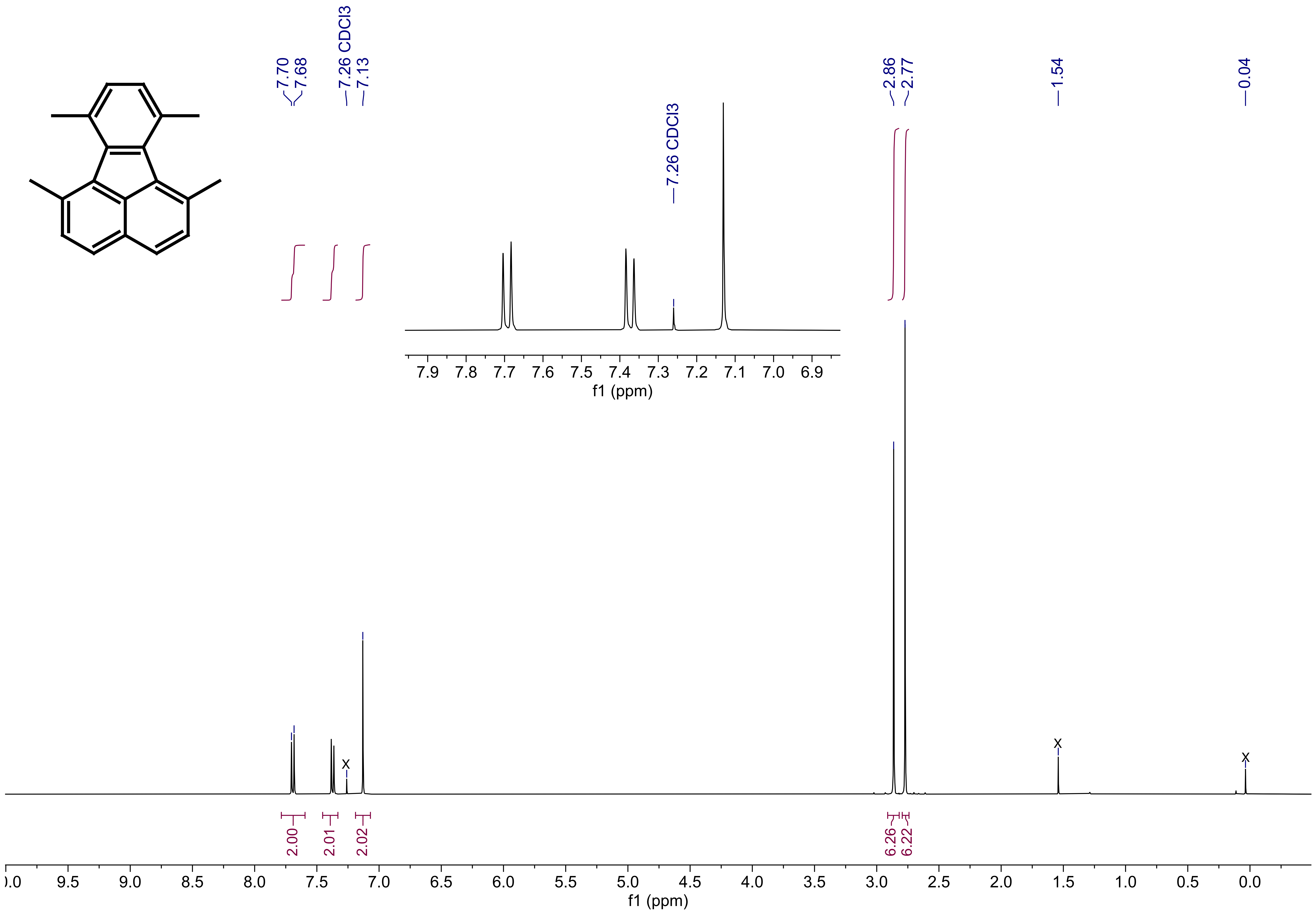

Supplementary Figure 14. Proton NMR of crude 4 in deuterated chloroform. Signals from TMS, water, and chloroform are shown with the help of a cross sign.

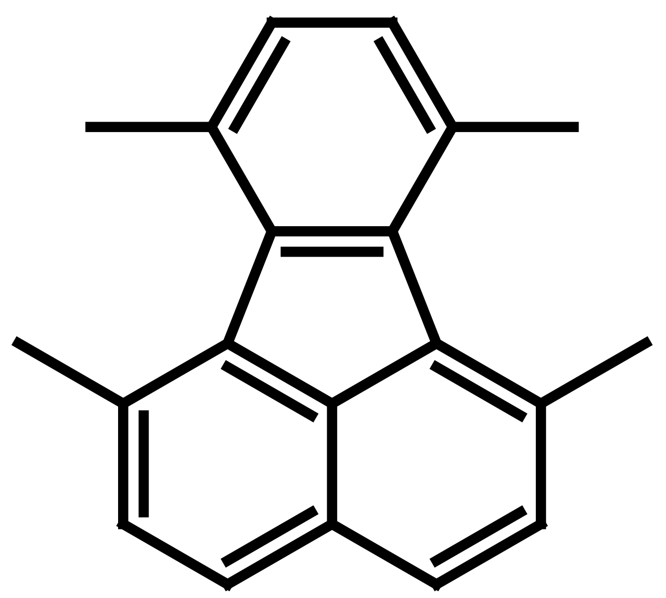

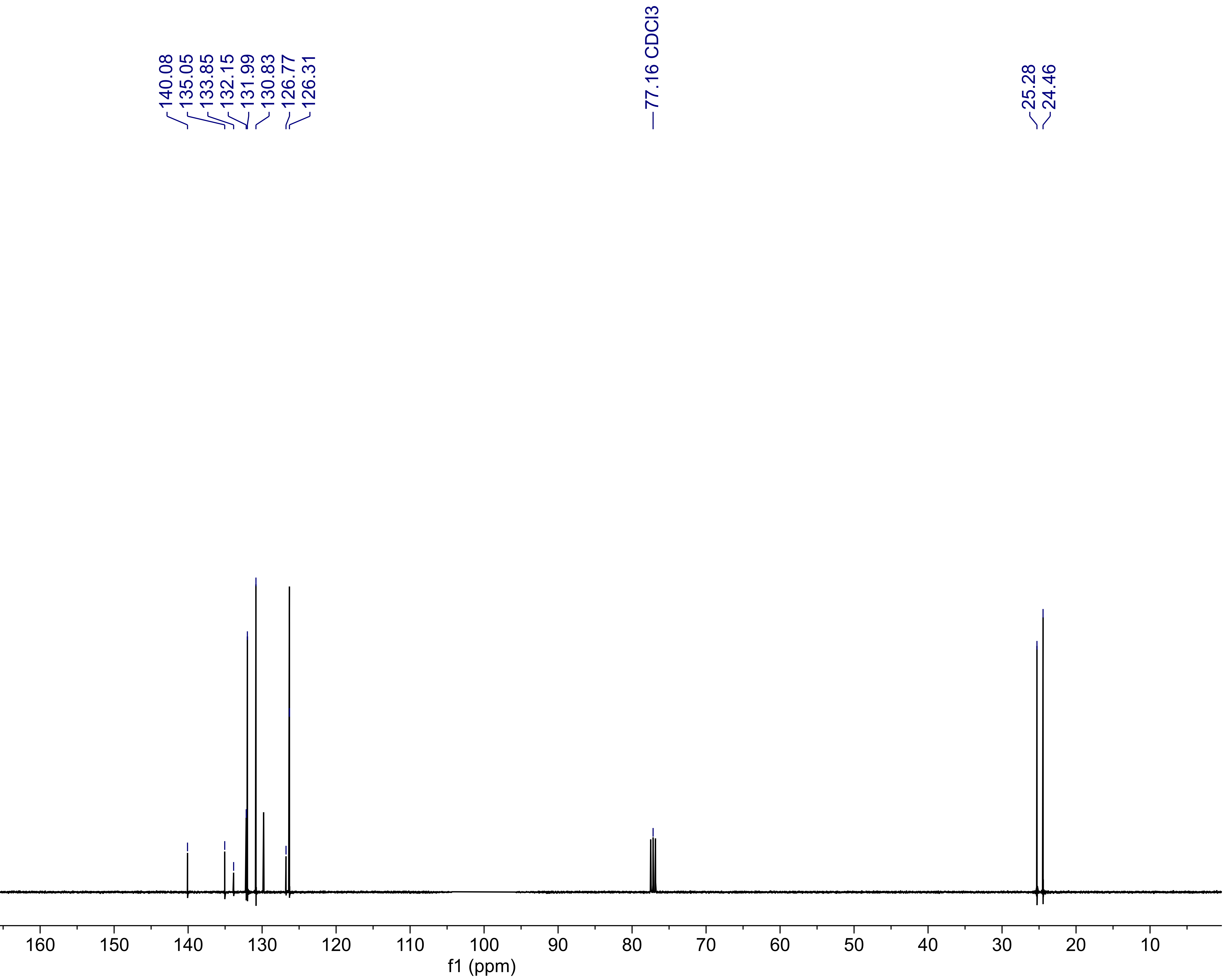

Supplementary Figure 15. Carbon-13 NMR of crude 4 in deuterated chloroform.

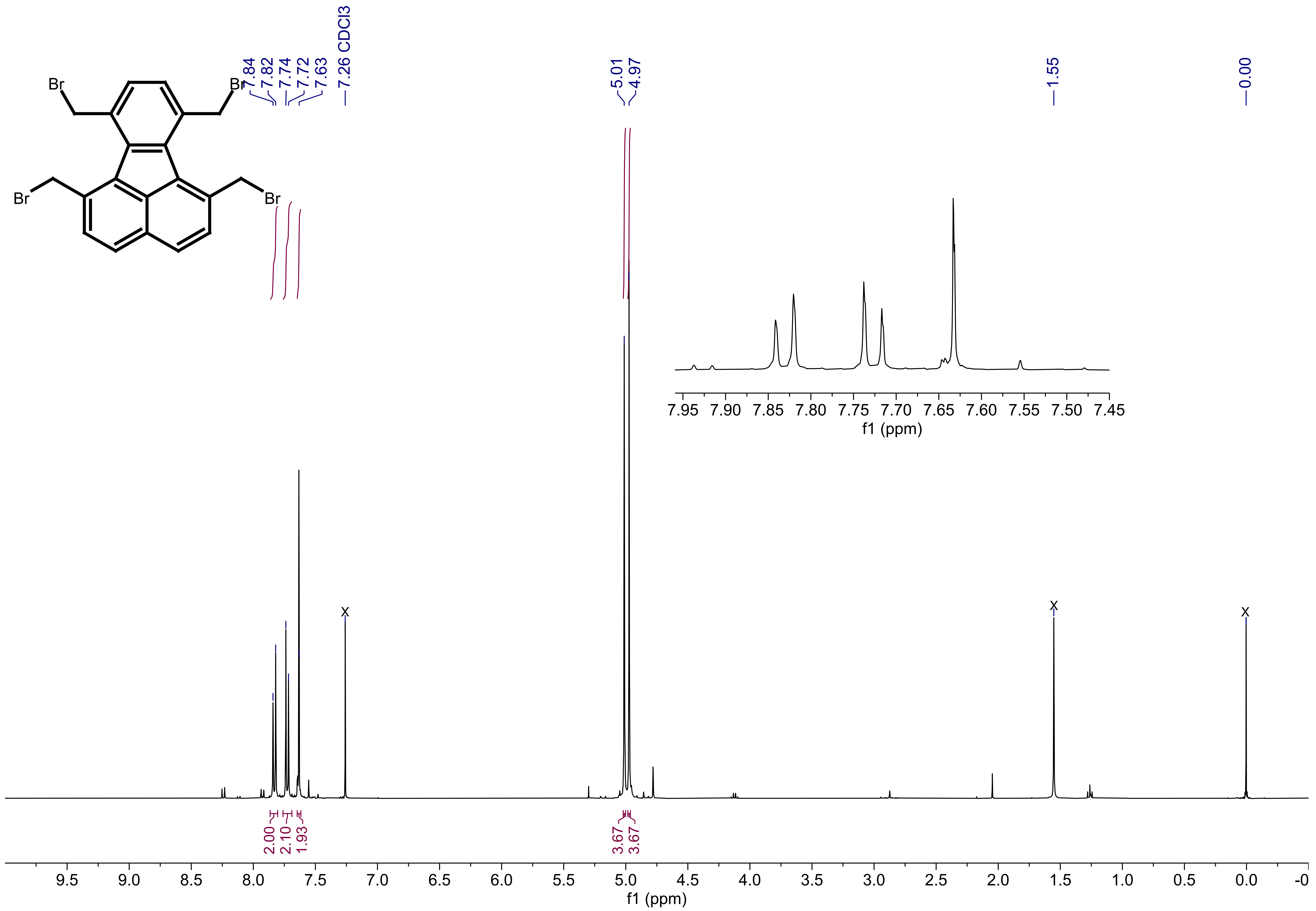

Supplementary Figure 16. Proton NMR of crude 1 in deuterated chloroform. Signals from TMS, water, and chloroform are shown with the help of a cross sign.

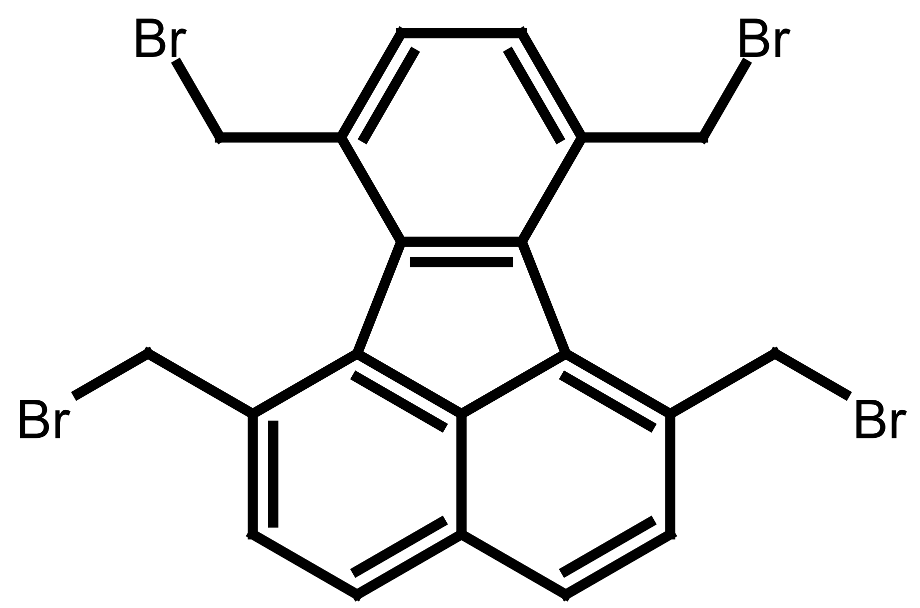

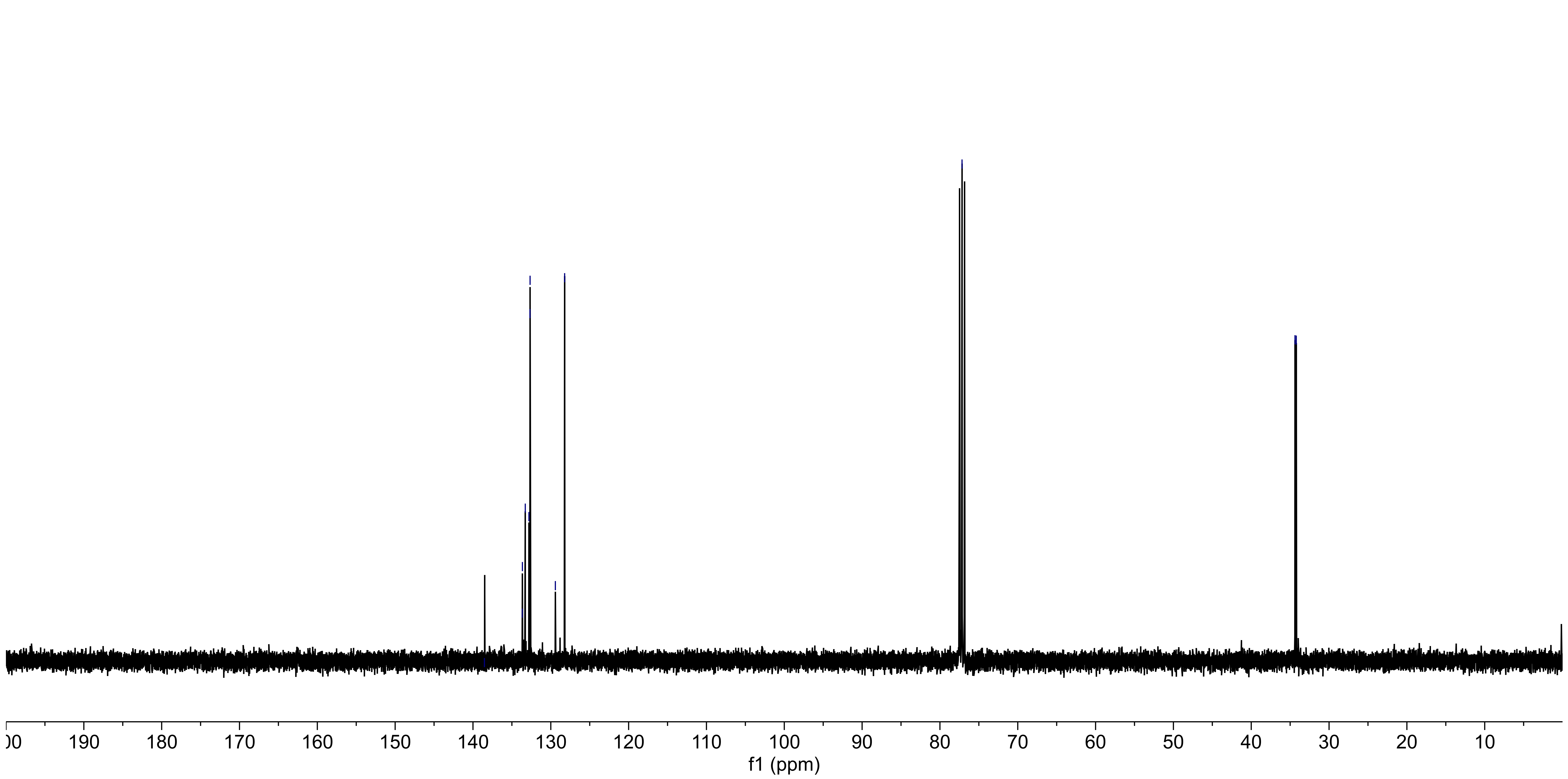

Supplementary Figure 17. Carbon-13 NMR of crude 1 in deuterated chloroform.

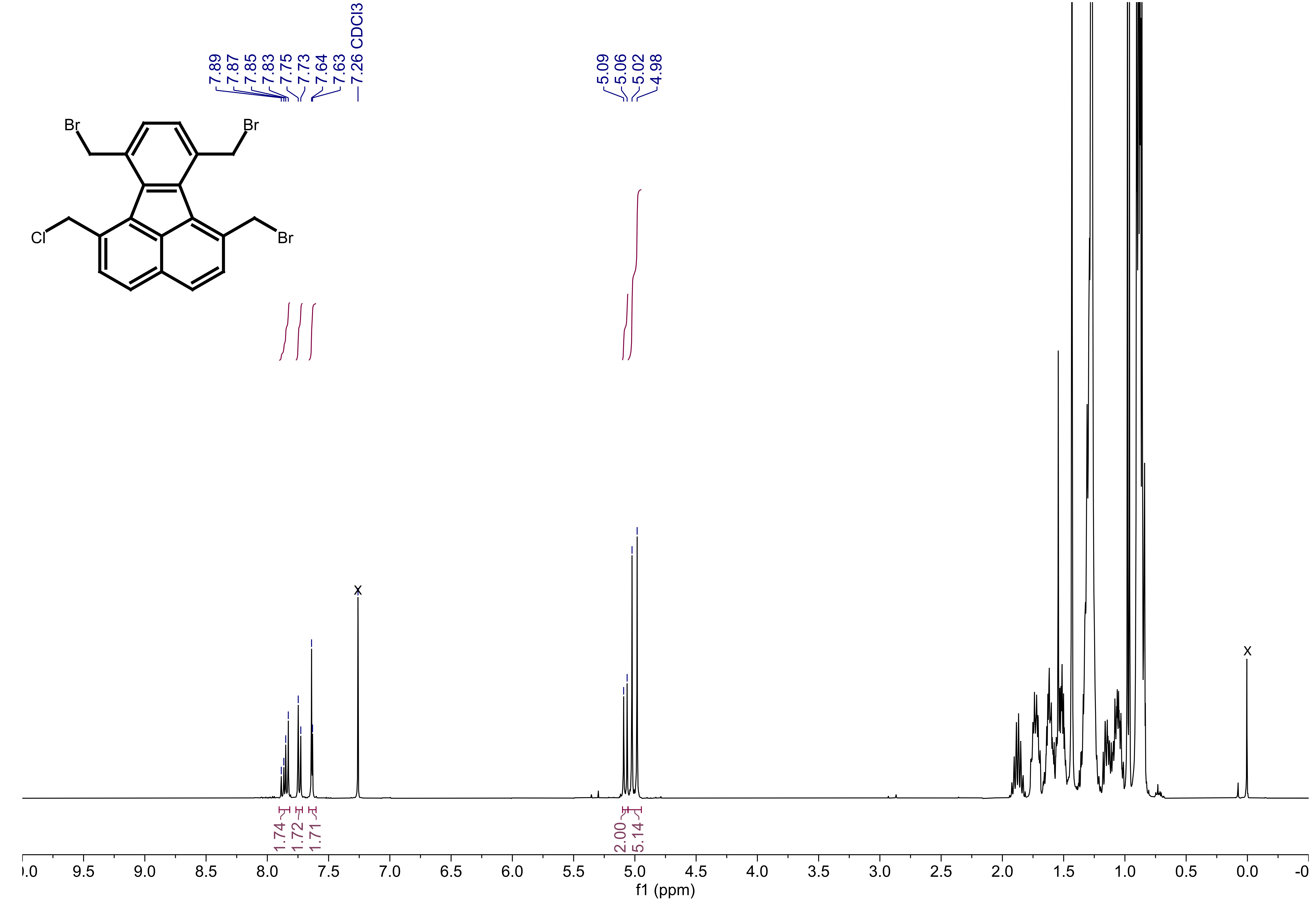

Supplementary Figure 18. Proton NMR of crude 6 in deuterated chloroform. Signals from TMS and chloroform are shown with the help of a cross sign.

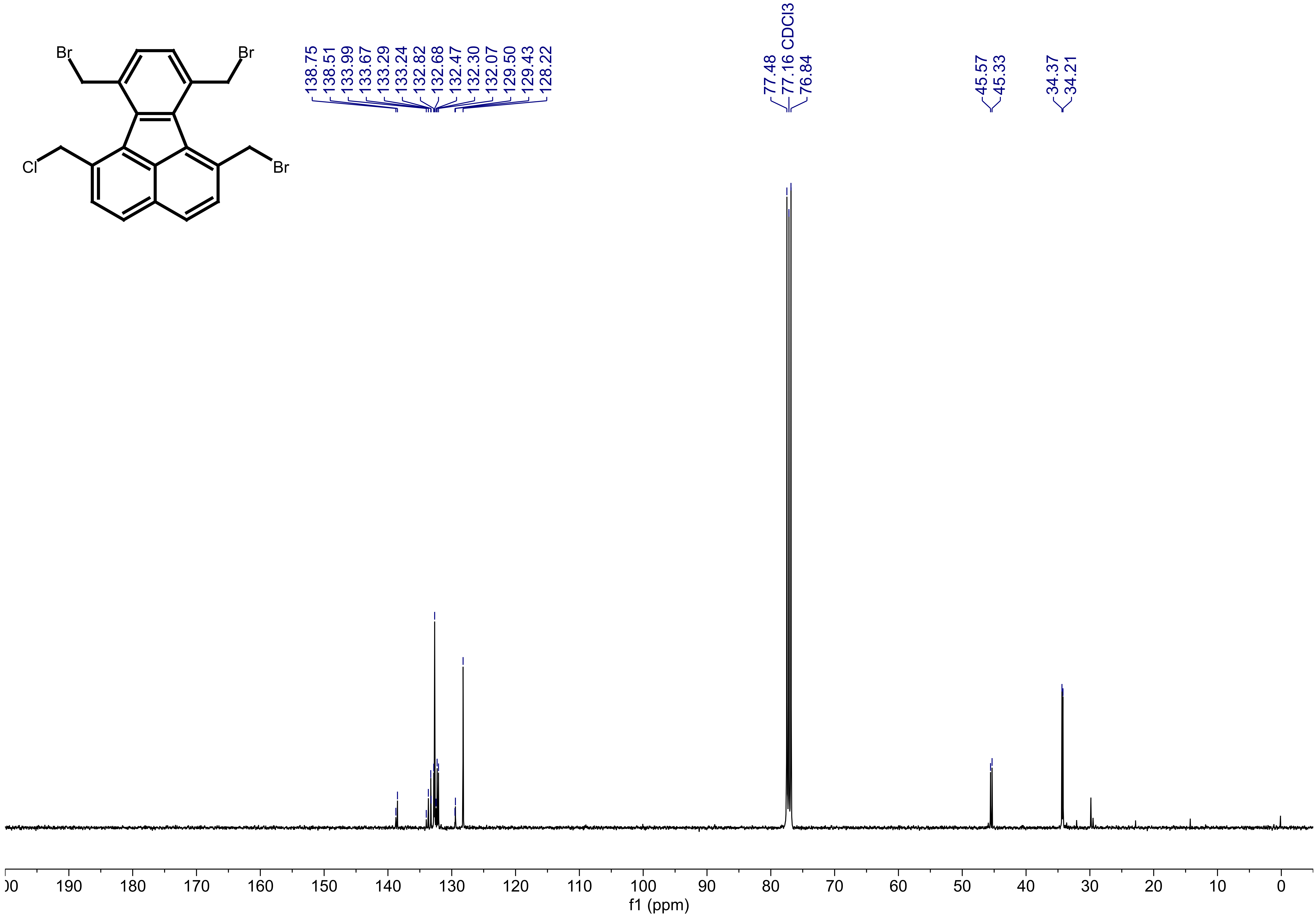

Supplementary Figure 19. Carbon-13 NMR of crude 6 in deuterated chloroform.

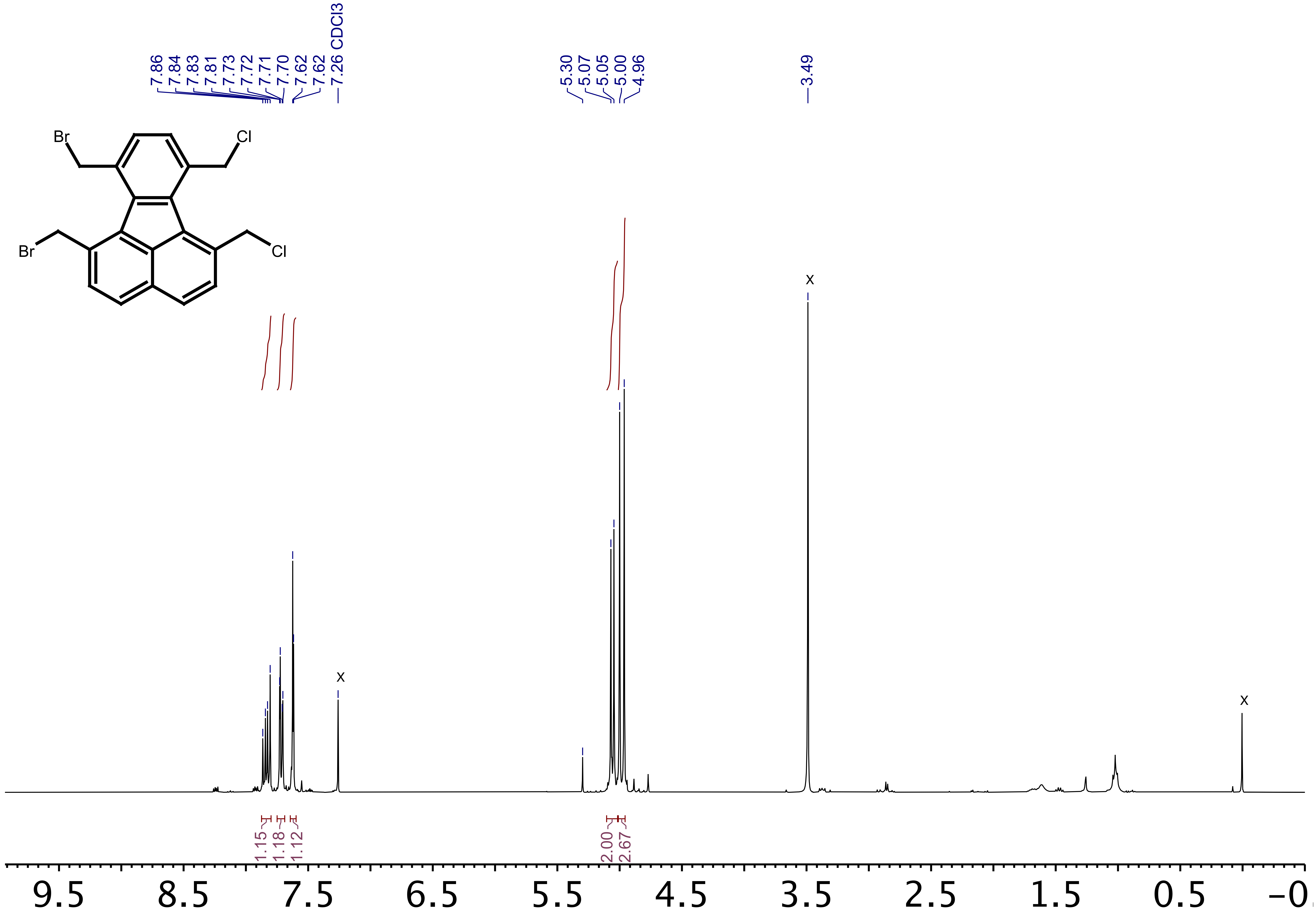

Supplementary Figure 20. Proton NMR of crude 5 in deuterated chloroform. Signals from TMS, methanol, and chloroform are shown with the help of a cross sign.

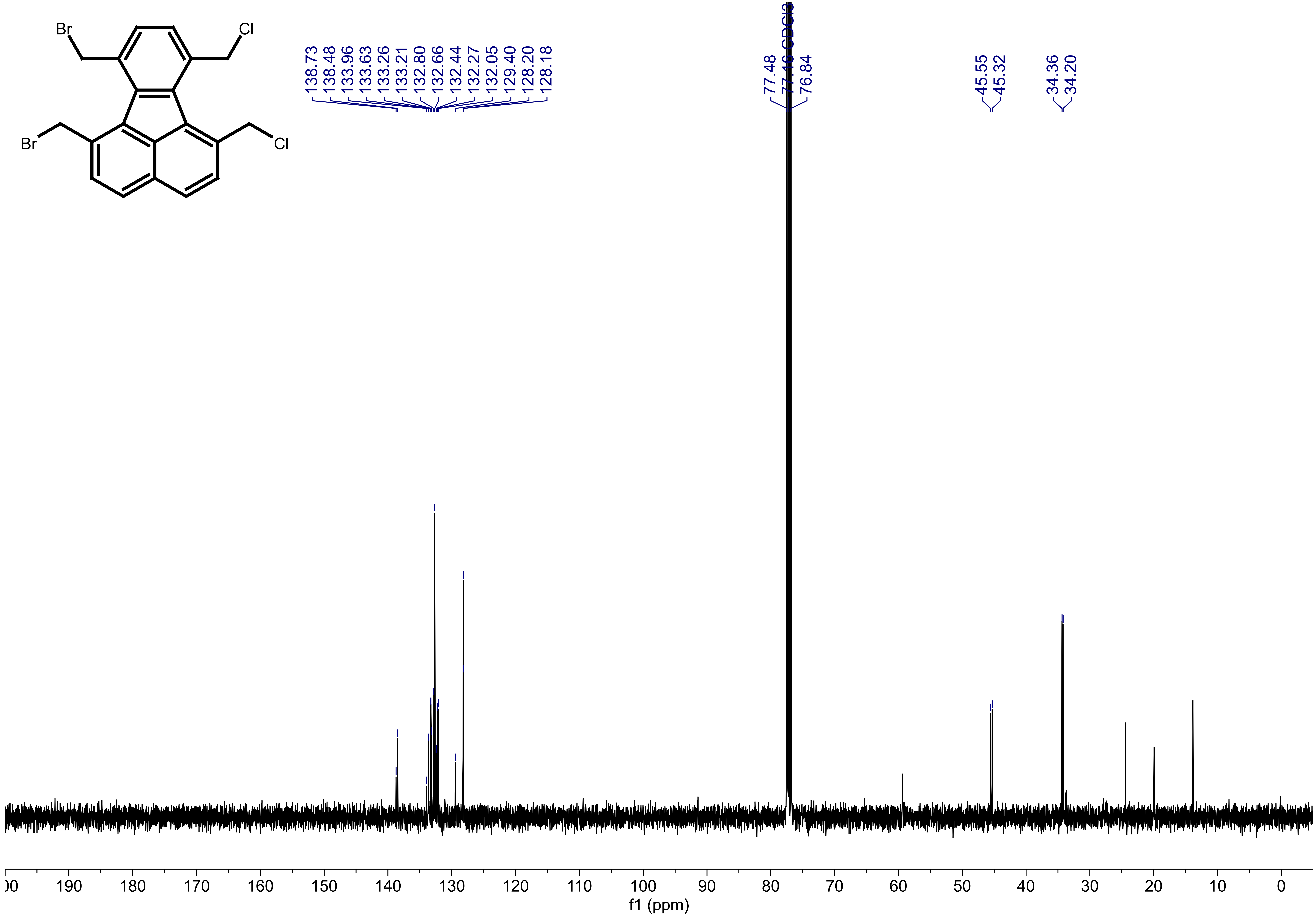

Supplementary Figure 21. Carbon-13 NMR of crude 5 in deuterated chloroform.

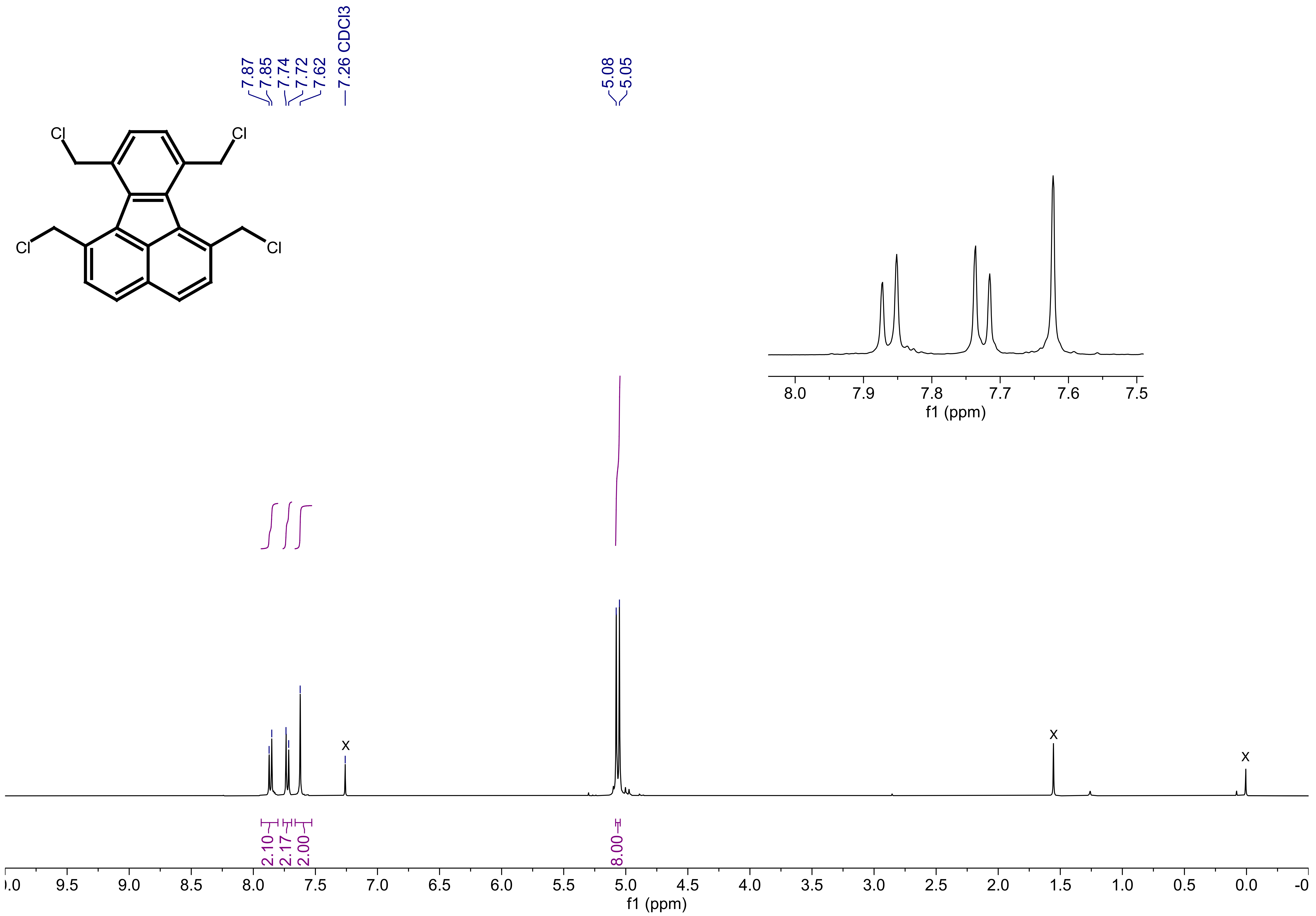

Supplementary Figure 22. Proton NMR of 8 in deuterated chloroform. Signals from TMS, water, and chloroform are shown with the help of a cross sign.

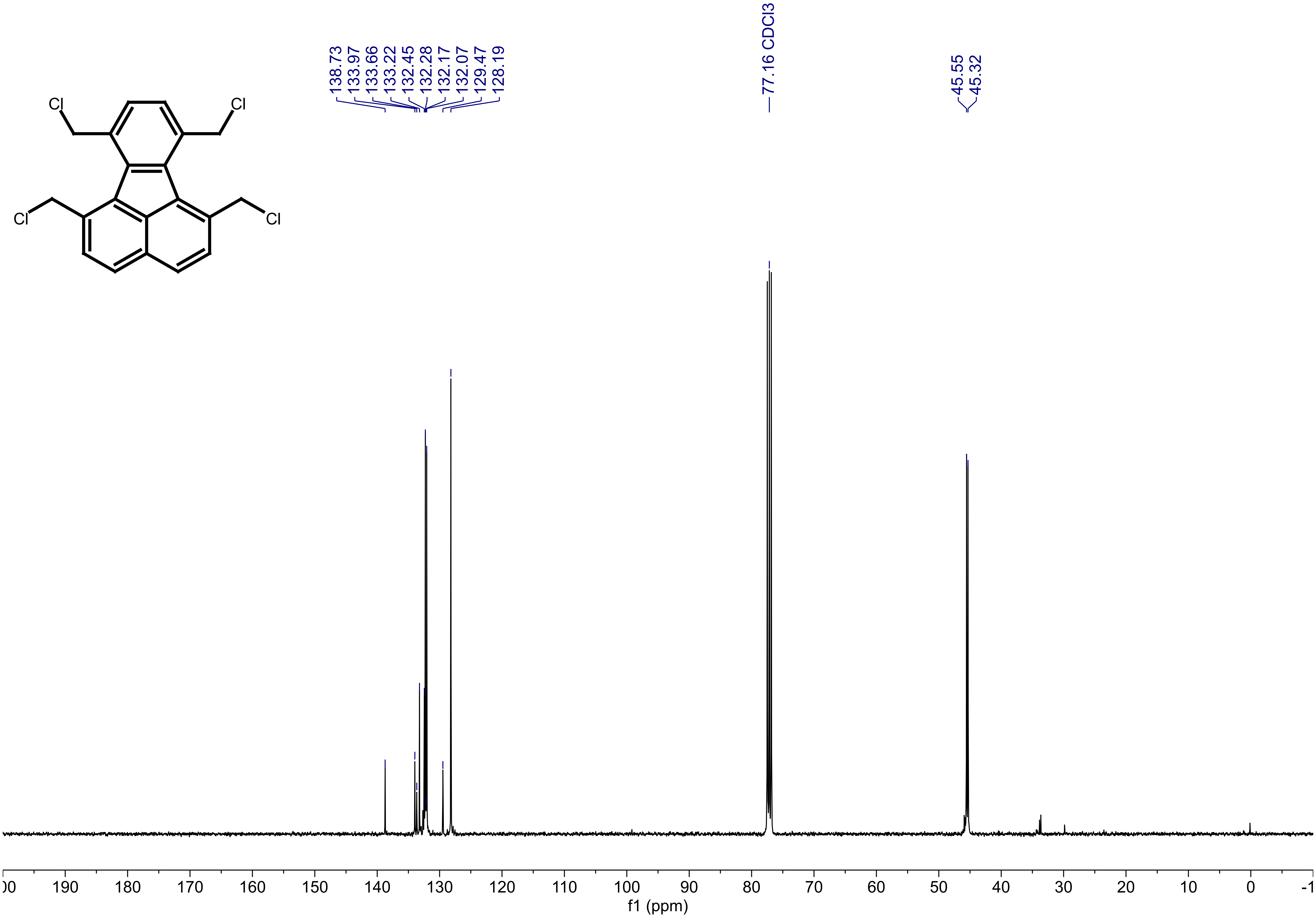

Supplementary Figure 23. Carbon-13 NMR of 8 in deuterated chloroform.

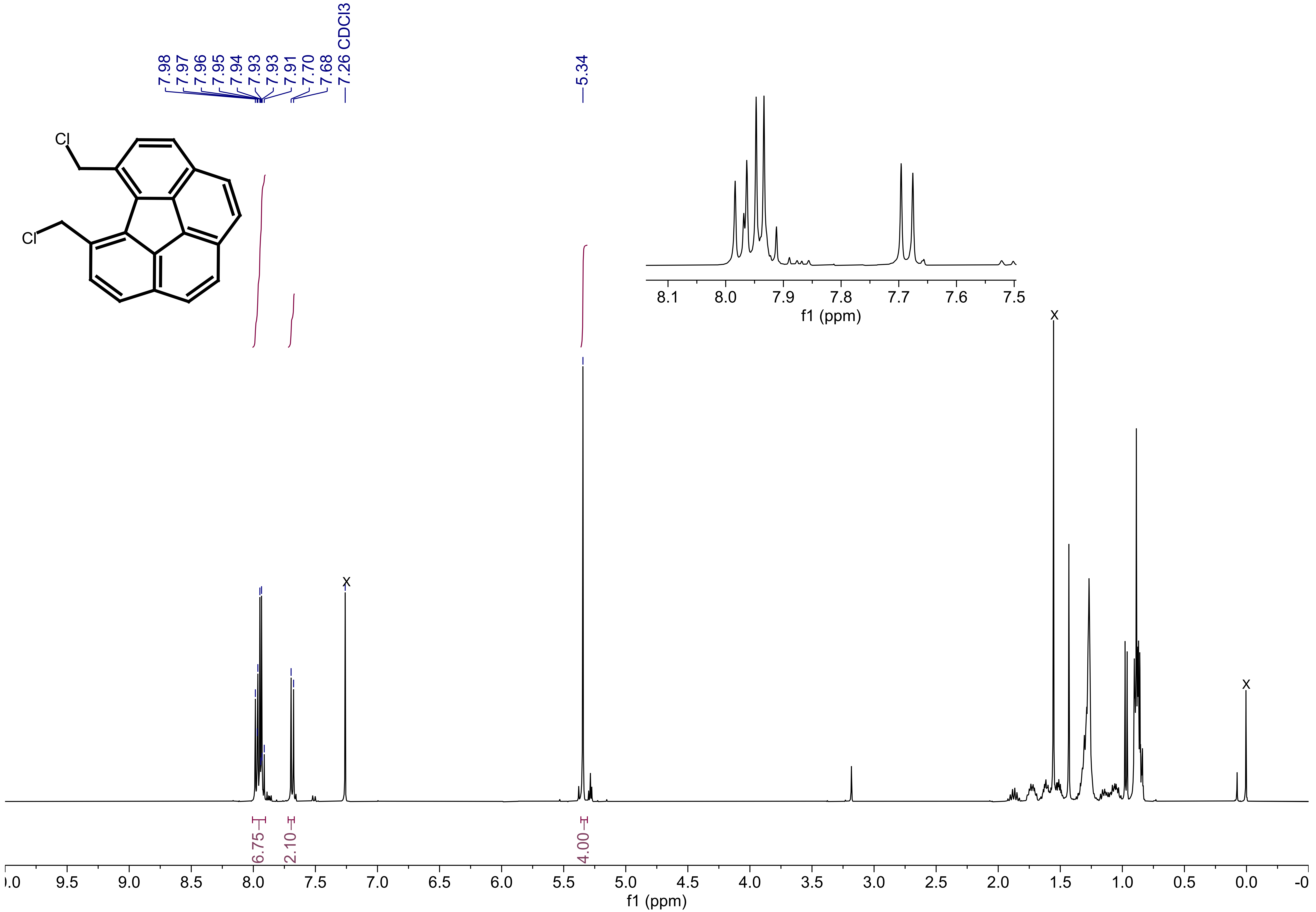

Supplementary Figure 24. Proton NMR of crude 7 in deuterated chloroform. Signals from TMS, water, and chloroform are shown with the help of a cross sign.

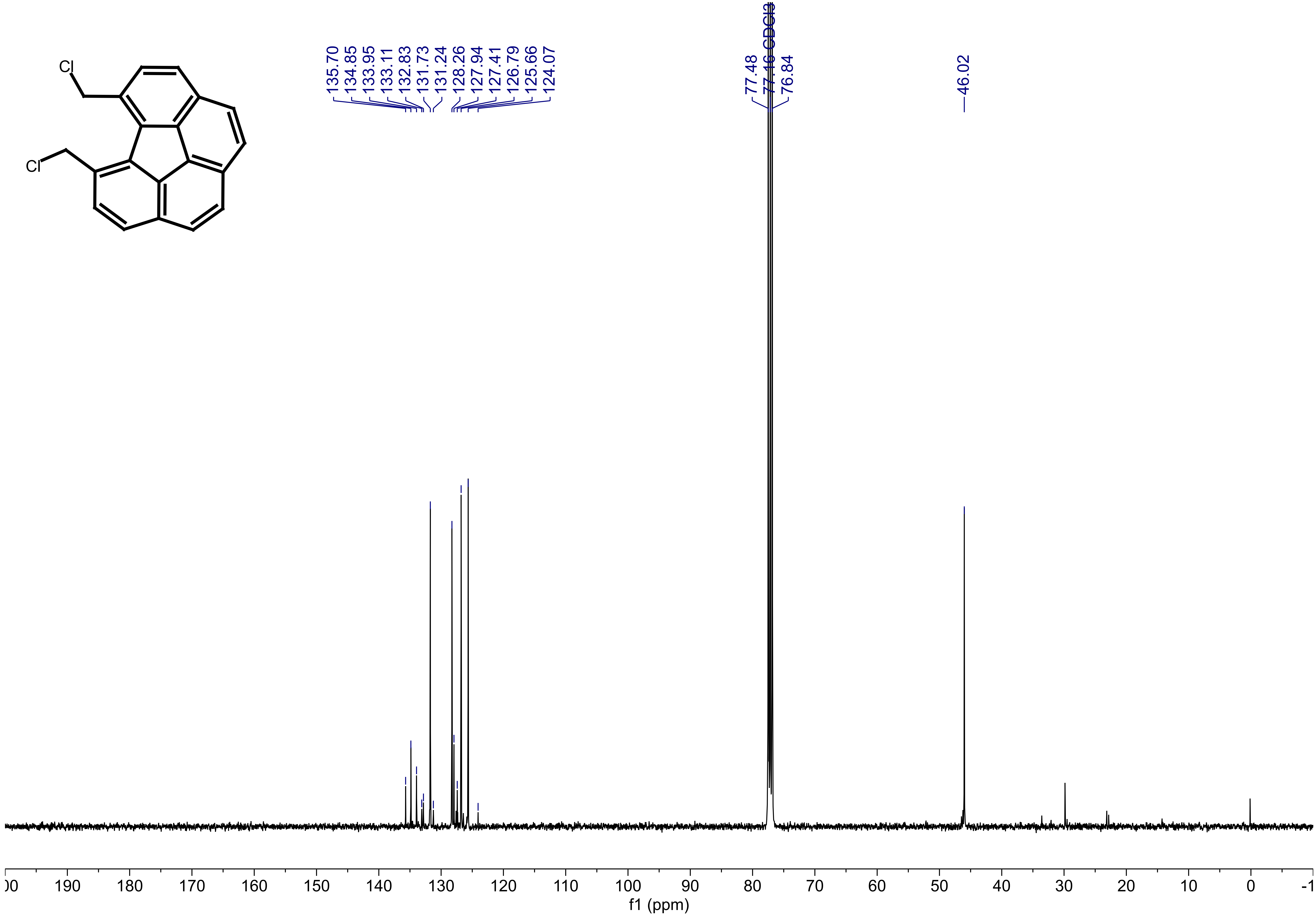

Supplementary Figure 25. Carbon-13 NMR of crude 7 in deuterated chloroform.

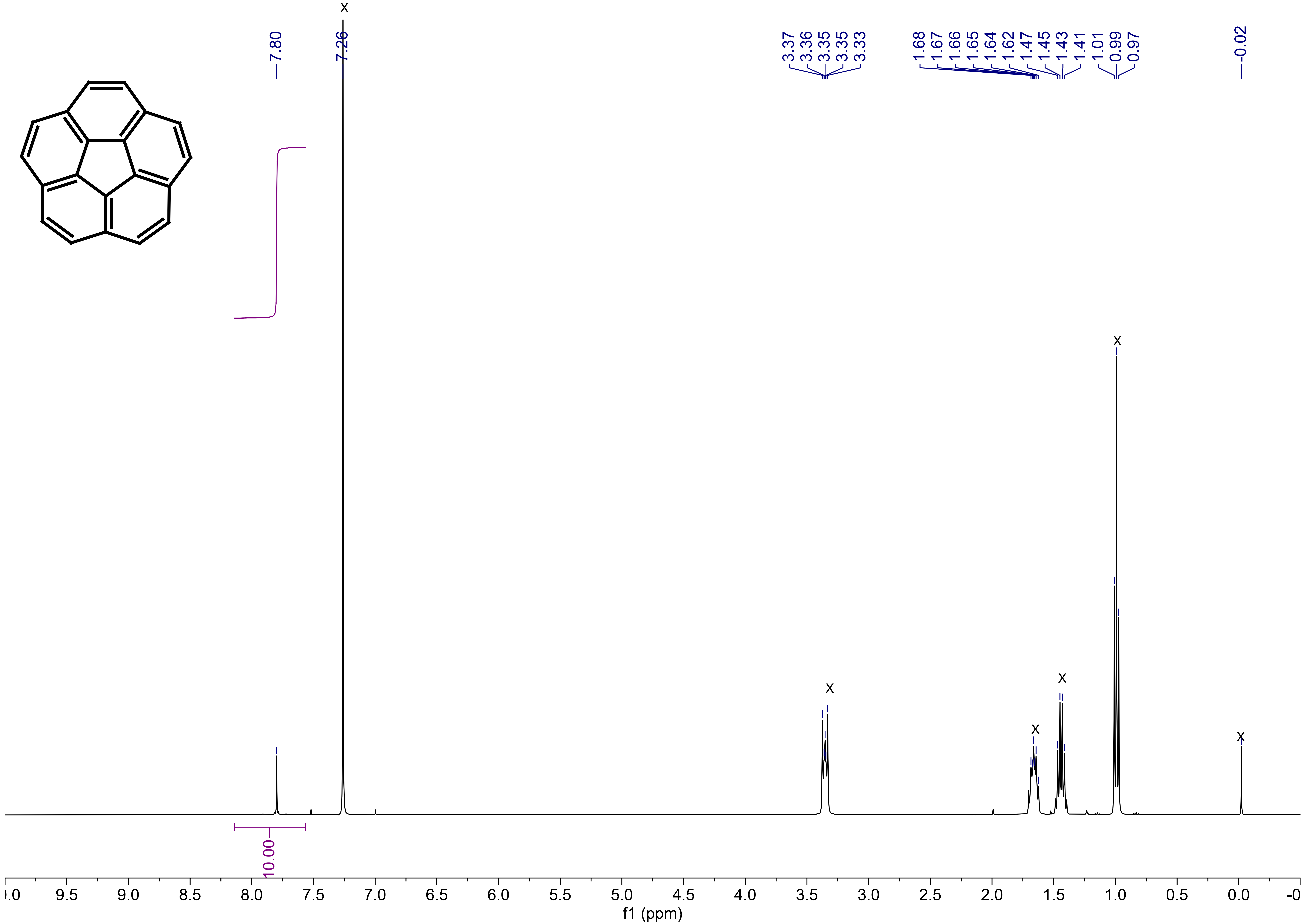

Supplementary Figure 26. Proton NMR of crude corannulene in deuterated chloroform. Signals from TMS, TBACl, and chloroform are shown with the help of a cross sign.

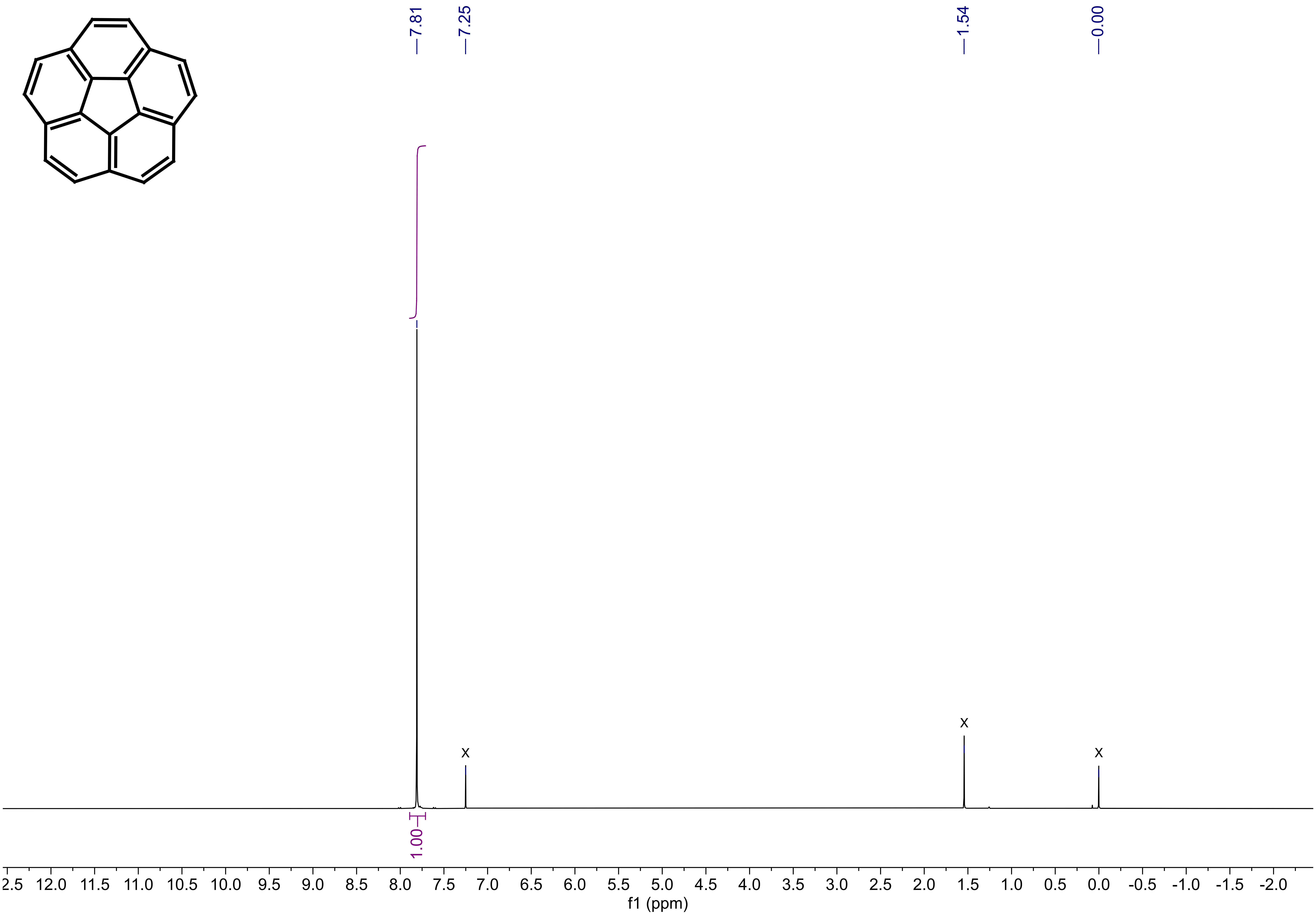

Supplementary Figure 27. Proton NMR of pure corannulene in deuterated chloroform. Signals from TMS, water, and chloroform are shown with the help of a cross sign.

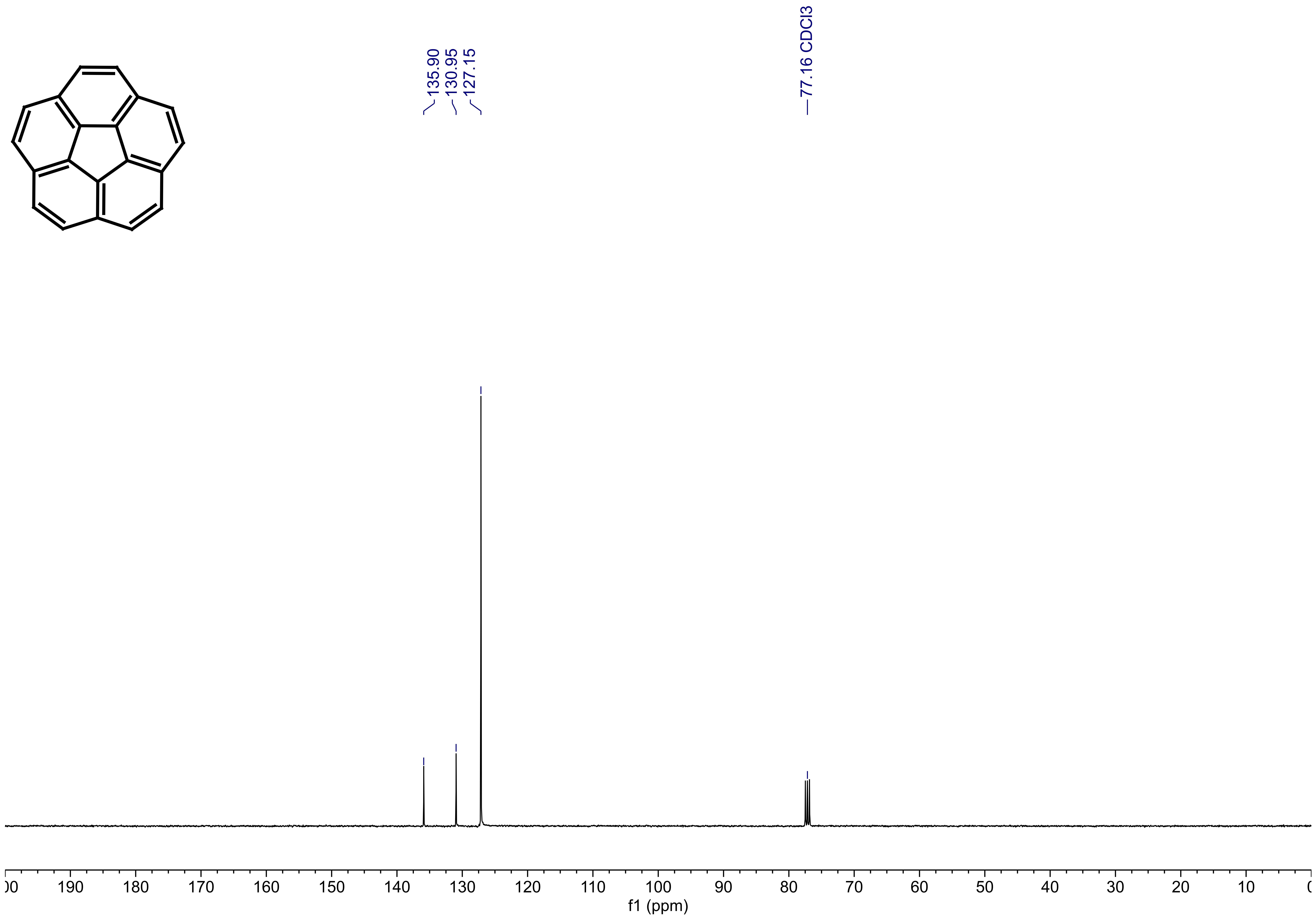

Supplementary Figure 28. Carbon-13 NMR of pure corannulene in deuterated chloroform.

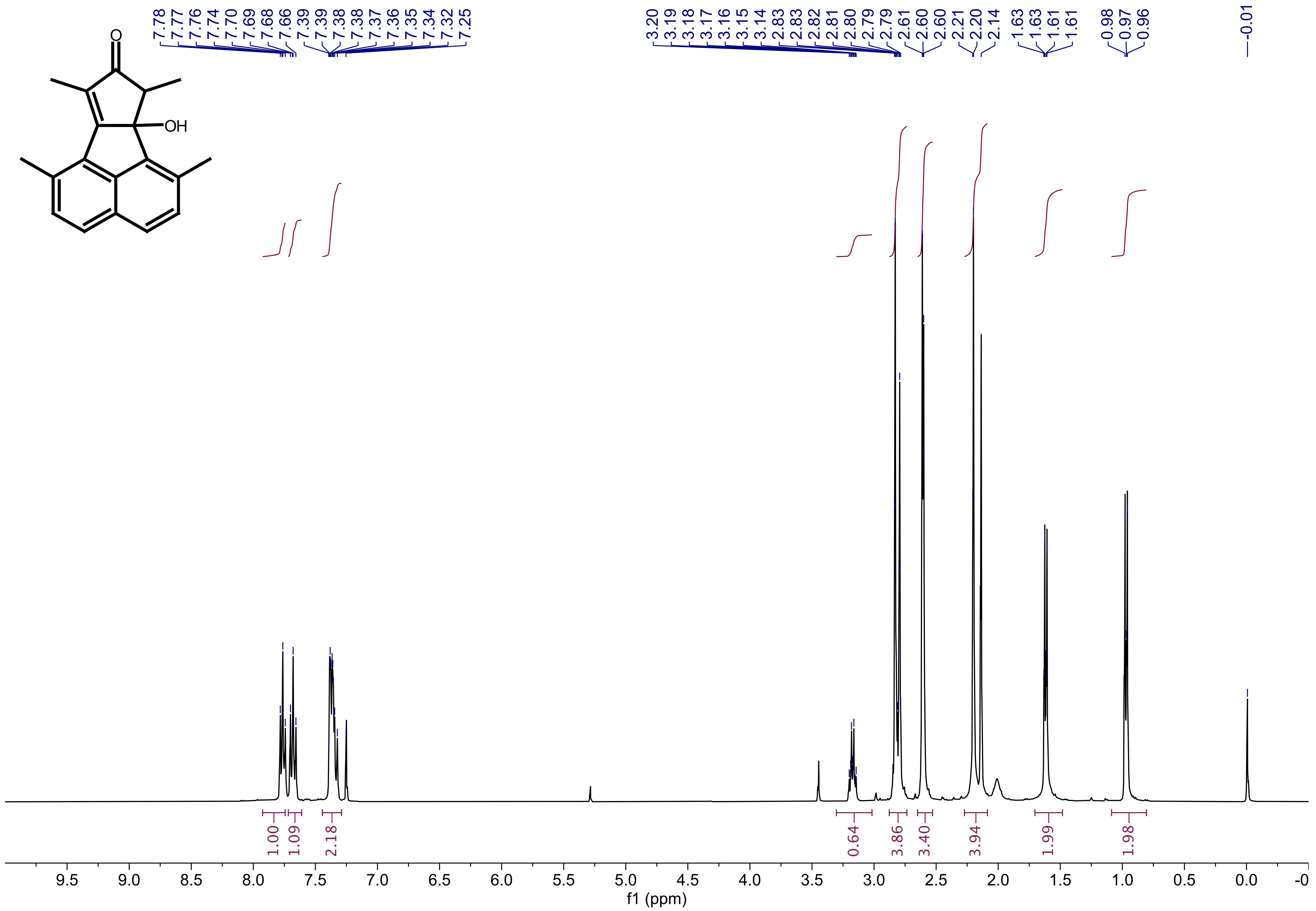

Supplementary Figure 29. Proton NMR of crude 3 from solution-phase synthesis in deuterated chloroform.

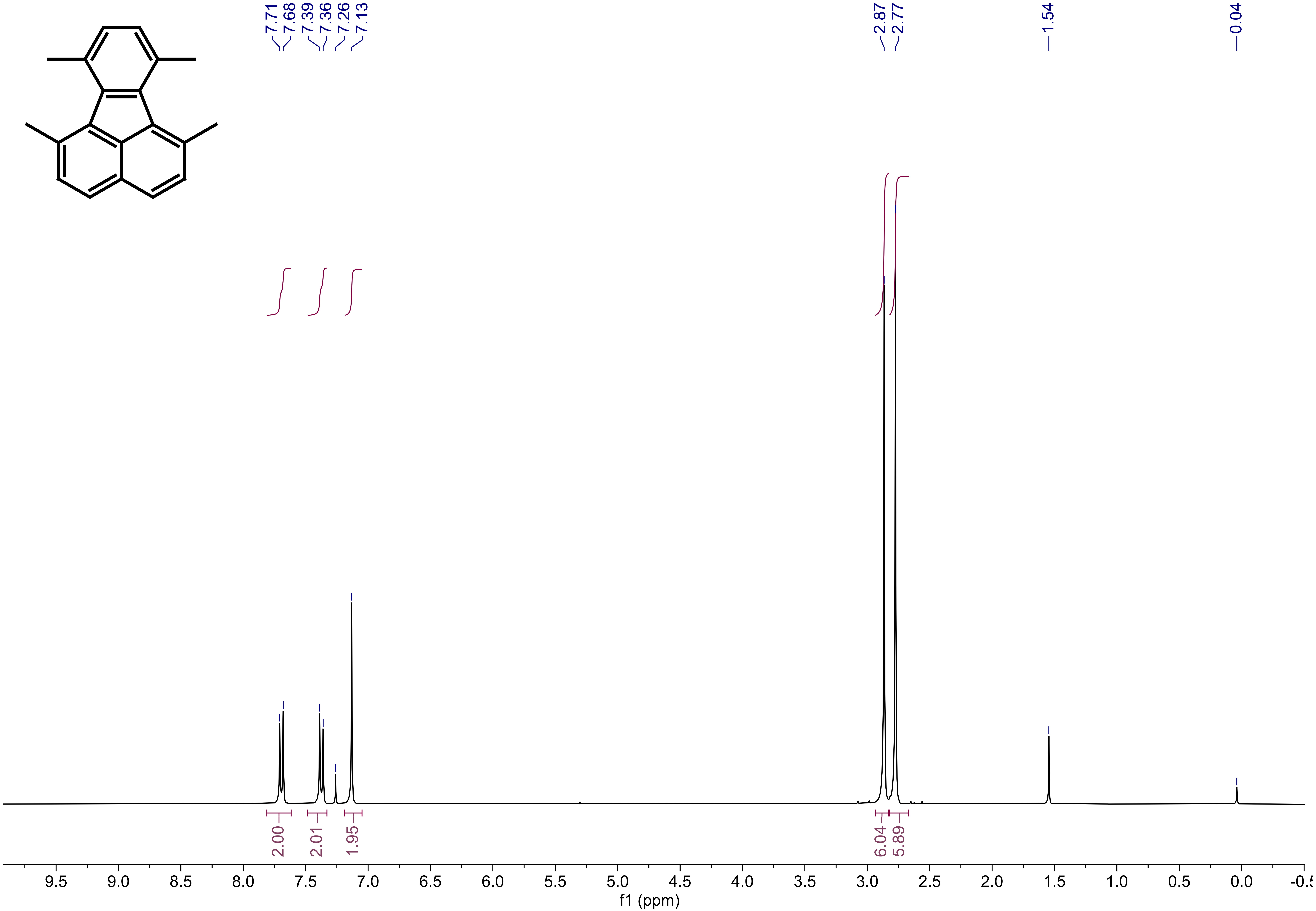

Supplementary Figure 30. Proton NMR of crude 4 from solution-phase synthesis in deuterated chloroform.

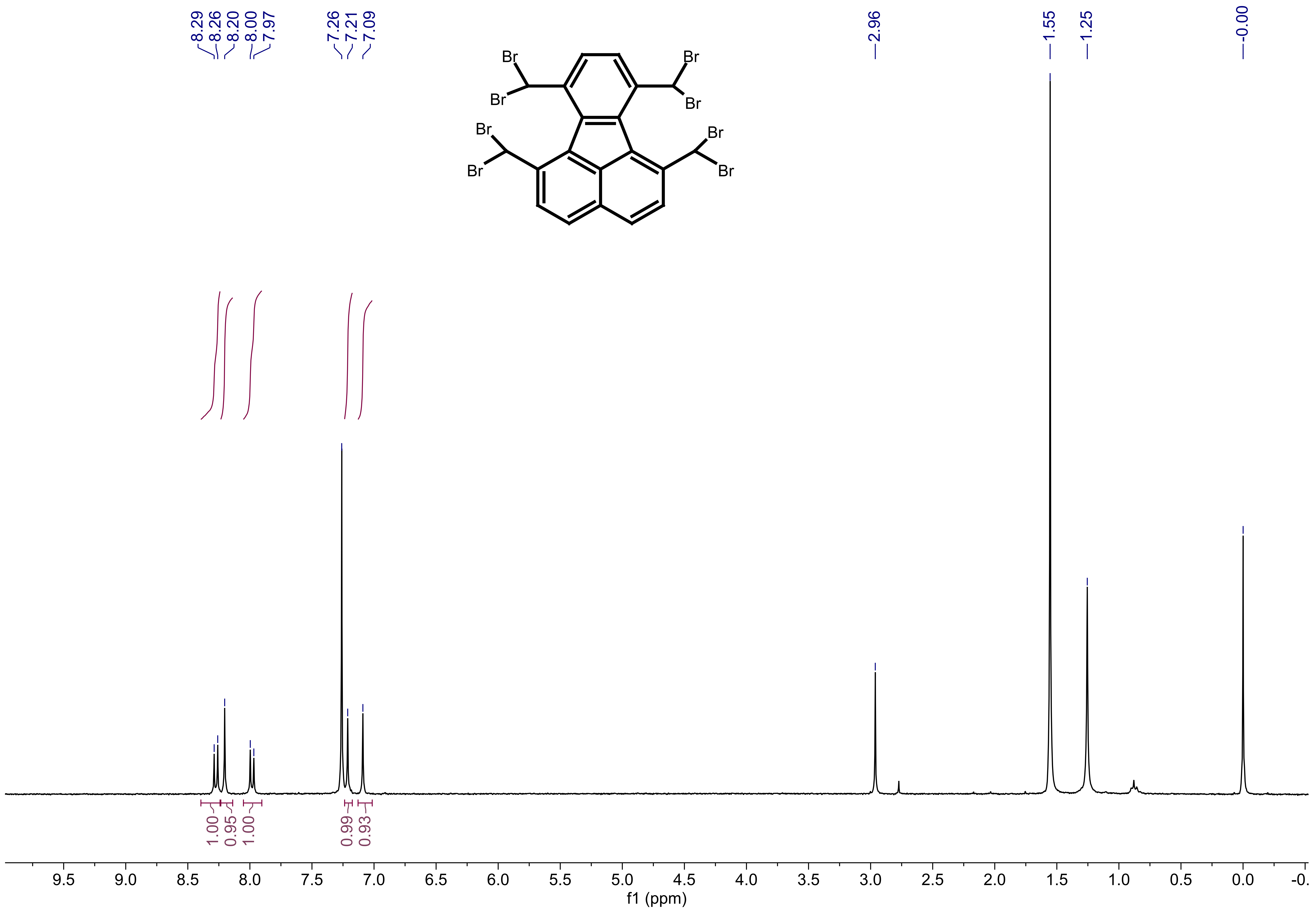

Supplementary Figure 31. Proton NMR of 9 from solution-phase synthesis in deuterated chloroform.

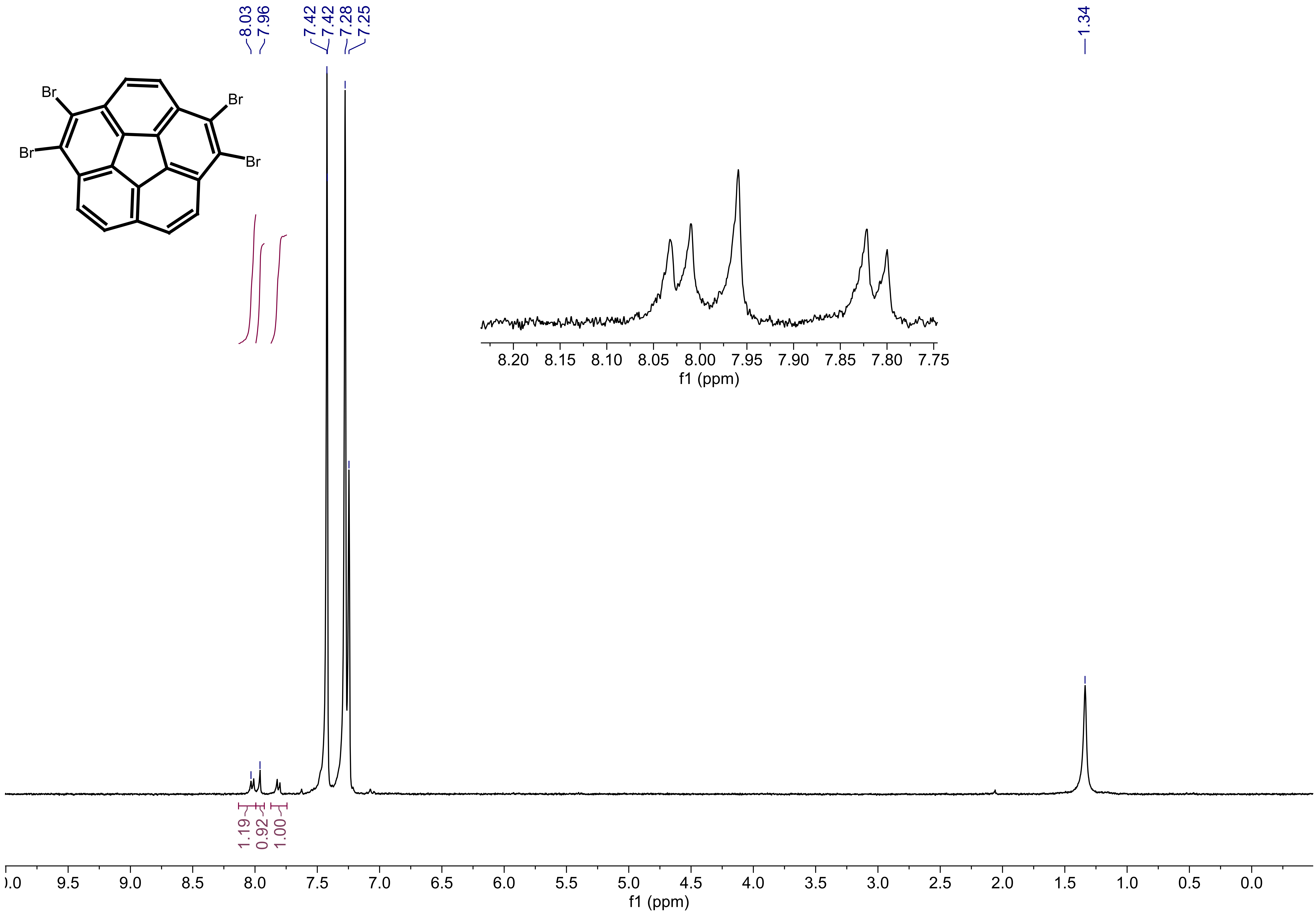

Supplementary Figure 32. Proton NMR of 10 from solution-phase synthesis in deuterated chloroform.
